# *Kazachstania pintolopesii* triggers an immune-endothelial-fibroblast cascade and drives inflammatory arthritis and tissue fibrosis in genetically susceptible hosts

**DOI:** 10.3389/fcimb.2025.1738184

**Published:** 2025-12-11

**Authors:** Haiting Zhang, Lei Li, Duanling Tan, Chulan Lin, Yanqing Zhang, Diling Chen

**Affiliations:** 1 Traditional Chinese medicine department, the Affiliated Guangdong Second Provincial General Hospital of Jinan University, Guangzhou, Guangdong, China; 2 School of Traditional Chinese Medicine, Jinan University, Guangzhou, Guangdong, China; 3 Research and Development Department, Guangdong Yier Biotechnology Co., LTD, Guangzhou, Guangdong, China

**Keywords:** autoimmunity, inflammatory arthritis, *Kazachstania pintolopesii*, multicellular crosstalk, tissue fibrosis

## Abstract

Emerging evidence underscores the critical role of microbiota dysbiosis in autoimmune pathogenesis, yet direct links between specific microbial species and disease mechanisms remain poorly defined. Our prior work identified *Kazachstania pintolopesii*, a gut-dwelling fungus isolated from spontaneous ankylosing spondylitis (AS)-prone monkeys, as a potent inducer of PANoptosome assembly. However, the multicellular and molecular mechanisms underlying its pathogenicity remained elusive. Here, we elucidate how lysates of *K. pintolopesii*(LKP) disrupt immune homeostasis through immune-endothelial-fibroblast crosstalk, metabolic reprogramming, and dysregulated cytokine networks in genetically susceptible hosts. Using single-nucleus RNA sequencing and functional assays, we demonstrate that LKP triggers robust inflammatory arthritis and tissue fibrosis in *BALB/c ZAP70W163C*mutant mice. Results showed that LKP injection induces severe joint destruction, spinal deformities, and upregulation of pro-inflammatory cytokines (IL-1β, IL-6, NF-κB) in joint tissues; immune-endothelial-fibroblast networks are dysregulated, with T cells promoting osteoclastogenesis via ligand-receptor interactions (e.g., *Sema4d-Plxnb1*) and endothelial cells exhibiting impaired migratory capacity and glycolytic reprogramming; fibroblast-like synoviocytes (FLS) undergo abnormal proliferation, with subpopulations (e.g., Fib_*Cmss1*, Fib_*Tnc*) driving extracellular matrix remodeling through TGF-β/PI3K-Akt signaling; and distinct macrophage subtypes (e.g., Mac_*Adam8*, Mac_*mt-Col*) exhibit ferroptosis and PI3K-Akt activation, contributing to osteoclastogenesis and cartilage degradation. Mechanistically, LKP disrupts mitochondrial function, enhances IL-17/TNF-α signaling, and induces pan-inflammatory responses in genetically predisposed hosts. Therapeutic targeting of these pathways (e.g., IL-17/IL-6 inhibitors, metabolic modulators) may disrupt the pathogenic cascade. Our findings establish *K. pintolopesii* as a keystone pathobiont in autoimmune arthritis and fibrosis, offering actionable insights for precision medicine.

## Introduction

Rheumatoid diseases encompass a group of chronic autoimmune disorders primarily affecting the joints, characterized by pain, swelling, stiffness, and progressive joint destruction (Wang et al., 2022). Accumulating evidence underscores the importance of host microbiota, including communities in the gut, oral cavity, skin, and respiratory tract, in the pathogenesis of these conditions (Kayama et al., 2020; Ruff et al., 2020; Yang and Cong, 2021; Cheng et al., 2022; Zhao et al., 2022). Alterations in microbial composition have been consistently observed in patients with rheumatoid diseases (Kurilshikov et al., 2021; Zhao et al., 2022), suggesting that microbiota may influence disease development through mechanisms such as molecular mimicry, immune activation, and barrier disruption. Further research is essential to elucidate these complex host-microbe interactions and to explore microbiome-targeted therapeutic strategies.

Ankylosing spondylitis (AS) is a chronic inflammatory disease predominantly affecting the axial skeleton. Although its pathogenesis remains incompletely understood, the gut microbiota has emerged as a key contributor (Lobiuc et al., 2025; Wei et al., 2025). Studies have revealed dysbiosis in AS patients, with increased abundance of bacteria such as *Klebsiella pneumoniae* and other *Enterobacteriaceae* (Chu et al., 2021; Long et al., 2022). These microbes may provoke immune activation via molecular mimicry or other mechanisms. Similarly, alterations in the oral microbiota, including an association with *Porphyromonas gingivalis*, have been reported in AS, potentially linking periodontal inflammation to disease initiation or progression (Lv et al., 2021; Kim et al., 2025). Additionally, a history of infections (e.g., with *Chlamydia pneumoniae* or *Klebsiella pneumoniae*) may trigger or exacerbate AS by activating immune pathways and promoting pro-inflammatory cytokine production, leading to inflammation and bone erosion (Feng et al., 2011; Zhang X. et al., 2021).

In rheumatoid arthritis (RA), gut dysbiosis is frequently observed, characterized by elevated *Prevotella copri* and reduced *Faecalibacterium prausnitzii* levels (Zaiss et al., 2021; Zhao et al., 2022). These shifts may influence immune function through microbial metabolites such as short-chain fatty acids (SCFAs), which modulate immune cell differentiation and activity. The oral microbiome also plays a role: patients with RA exhibit a higher prevalence of periodontal disease, and *Porphyromonas gingivalis*, which produces citrullinating enzymes, may promote autoantibody generation targeting joint tissues (Perricone et al., 2019; Li et al., 2022). Skin microbial alterations (e.g., *Staphylococcus aureus* overgrowth) and respiratory infections have further been associated with RA (Sams et al., 2015; Dieperink et al., 2024), suggesting multifaceted microbiota-immune interactions across different host sites. Interventions targeting the microbiome, including dietary modulation, probiotics, and fecal microbiota transplantation, hold promise as novel therapeutic avenues for RA (Gioia et al., 2020; Yu et al., 2021; Nikiphorou and Philippou, 2023; Yang et al., 2024).

Fungal infections have also been implicated in autoimmune diseases, including rheumatoid disorders and RA. For example, *Kazachstania pintolopesii* has been investigated in the context of polymicrobial interactions and immune regulation (Zhang et al., 2022; Liao et al., 2024). In macrophage-depleted mice, co-colonization with *Klebsiella pneumoniae*, *Enterococcus faecalis*, and *Acinetobacter radioresistens* was associated with worsened sepsis severity (Rheinlander et al., 2018; Roe, 2021; Lionakis et al., 2023; Javidnia et al., 2024), potentially amplified by *K. pintolopesii* induced cytokine production in enterocytes (Zhang et al., 2022). This suggests that macrophages may serve as a primary defense against this fungus. Our previous work demonstrated that *K. pintolopesii* lysate, derived from a spontaneous AS monkey model, triggers PANoptosome formation in RAW264.7 cells, though the underlying mechanism requires further elucidation (Zhang et al., 2022).

The microbiota may exhibit antigenic mimicry of self-antigens, potentially triggering autoantibody production and initiating autoimmune responses. For instance, certain bacterial proteins share structural similarities with human proteins involved in joint inflammation, which can lead to an immune response targeting articular tissues (Potter et al., 2002; Iliopoulou et al., 2008; Hou et al., 2022; Lu et al., 2022). Moreover, microbiota-immune interactions play a crucial role in immune modulation, influencing the activity of immune cells and cytokine networks (Huang et al., 2024). Microbiota-induced immune dysregulation is increasingly implicated in the pathogenesis of rheumatoid diseases, as some commensal or pathogenic bacteria can stimulate the production of pro-inflammatory cytokines, exacerbate inflammation and contributing to joint damage (Yang and Cong, 2021; Zhao et al., 2022; Dong et al., 2023).

Despite these advances, numerous questions remain regarding the biological functions of *K. pintolopesii*, its mechanisms of action, and its ecological interactions. The pathogenesis of AS and other rheumatoid diseases is multifactorial, involving genetic susceptibility, immune dysregulation, microbial influences, and environmental factors. Continued research is needed to fully understand these complex processes and develop effective therapeutic strategies.

## Results

### Lysate of *Kazachstania pintolopesii* induces severe inflammatory response in the BALB/c ZAP70^W163C^ mutant mouse

**Figure 1:**
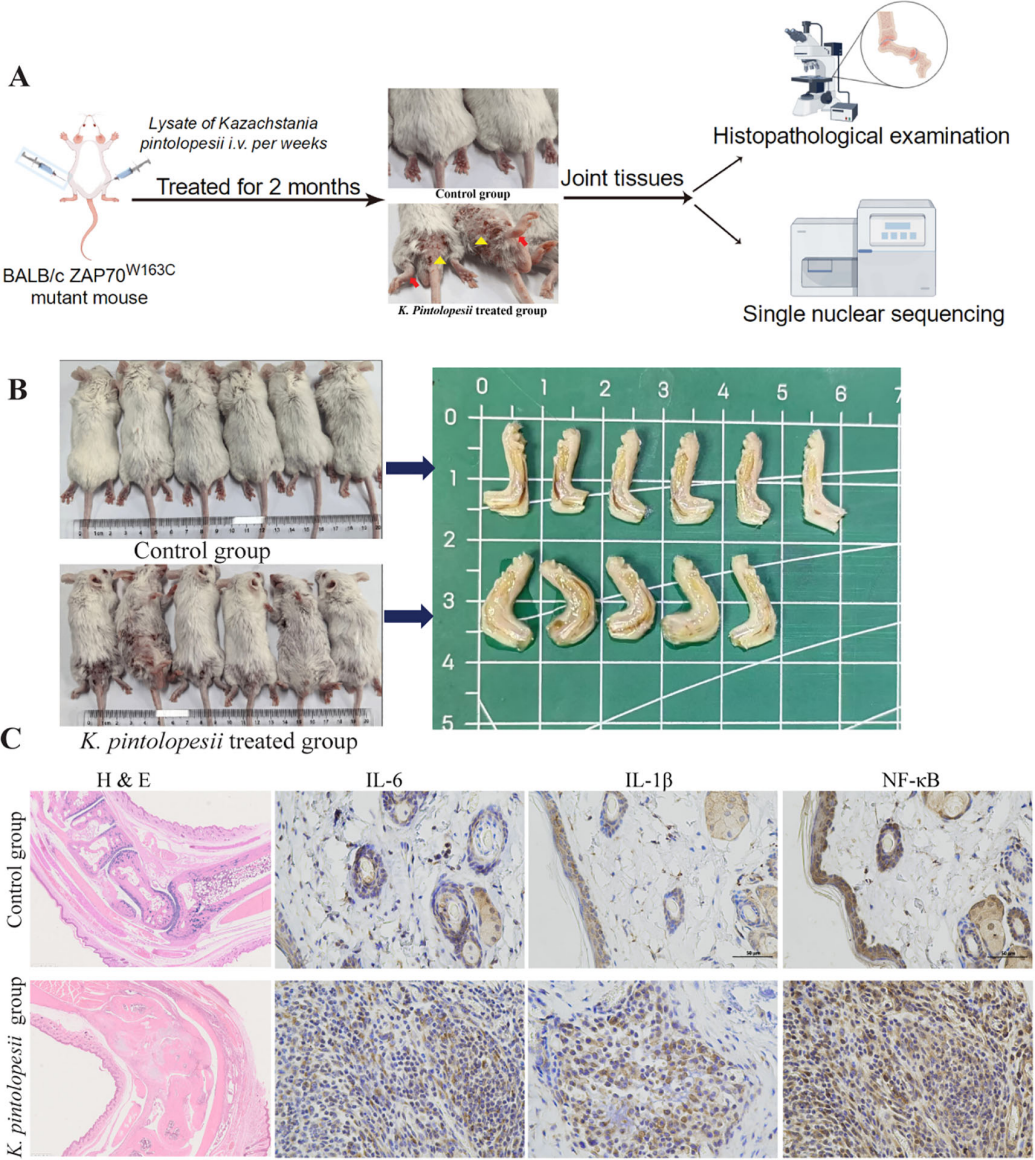
Lysate of *Kazachstania pintolopesii* induces severe inflammatory response in the BALB/c ZAP70^W163C^ mutant mouse. The experimental flow graph **(A)**; the external appearance of the mice showed reduced hair, ulcerated skin and swollen joints in the *K*. *pintolopesii* treated mice **(B)**; The pathological examination of the hind limbs of the *K*. *pintolopesii* treated mice **(C)**, and the expressions of IL-1β, IL-6 and NF-κB were sharply up-regulated in the *K*. *pintolopesii* treated mice **(C)**, and more details showed in **Supplementary Figure S1**. Data are presented as the means ± SD of 3 independent experiments.

To evaluate the pathogenic potential of *K. pintolopesii*, lysates of the fungus (LKP) were administered via direct injection into the paravertebral muscle tissues (**Figure 1A**). Consistent with its proposed pro-inflammatory role, LKP-treated mice exhibited more severe disease phenotypes, including skin ulceration, joint swelling, and spinal deformation, compared with the control group that the BALB/c ZAP70^W163C^ mutant mouse treated PBS (**Figure 1B**). Histopathological assessment of the hind limbs revealed structural disorganization in multiple articular regions: the talocrural, metatarsophalangeal, and interphalangeal joints showed blurred boundaries, with evident shrinkage or obliteration of the joint space (**Figure 1C**; **Supplementary Figure S1**). Inflammatory infiltrates and other pathological exudates were observed within the tibial cavity, and significant damage was also detected in the surrounding musculature (**Figure 1C**; **Supplementary Figure S1**). Furthermore, a marked upregulation of pro-inflammatory cytokines, including IL-1β, IL-6, and NF-κB, was detected in both spinal and joint tissues of LKP-treated ZAP70^W163C^ mutant mice (**Figure 1C**, p < 0.05). Collectively, these data demonstrate that *K. pintolopesii* lysate triggers autoimmune activation and robust inflammatory responses in the BALB/c ZAP70^W163C^ mouse model.

### Immune cell interaction network changed in the lysate of *K. pintolopesii* treated BALB/c ZAP70^W163C^ mutant mouse

**Figure 2:**
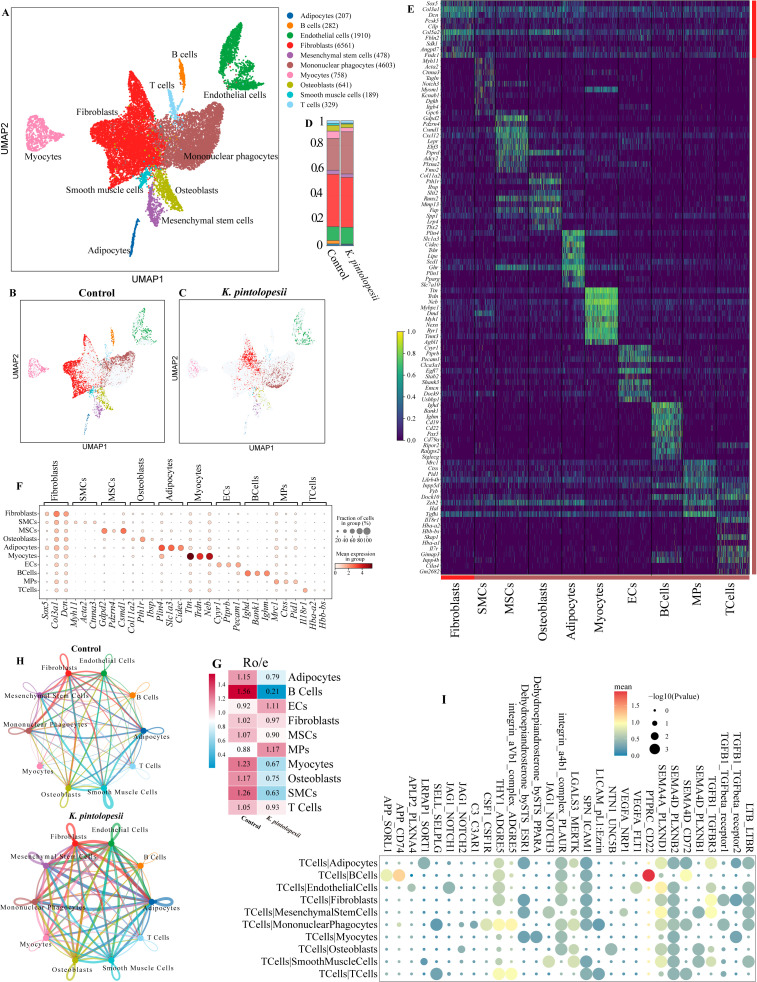
Immune cell interaction network changed in the Lysate of *K. pintolopesii* treated BALB/c ZAP70^W163C^ mutant mouse. The UMAP visualization illustrates the distribution of 10 major cell types **(A)**, along with their distinct spatial patterns in both the control **(B)** and *K*. *pintolopesii*-treated groups **(C)**. The proportional abundance of each cell type is quantified in **(D)**. Expression of cell-type-specific marker genes for all 10 cell types is depicted in **(E, F)**. The RO/E (ratio of observed to expected cell numbers) index was calculated to evaluate the enrichment or depletion of each cell type across conditions **(G)**. Cell-cell interaction analysis revealed a significant increase in communication network complexity within the *K*. *pintolopesii*-treated group **(H)**. Key ligand-receptor pairs mediating interactions between T cells and the other nine cell types are detailed in **(I)**, with further supporting data provided in **Supplementary Figure S2**.

In order to know the way of infection of *K. pintolopesii* to induced arthritis, we collected the joint tissue after the two months treatments of LKP for single nuclear sequencing. After quality control we collected a total of 15751 nuclear, covering 10 major cell types (**Figure 2A**), including Adipocytes (207), B cells (282), Endothelial cells (1910), Fibroblasts (6561), Mesenchymal stem cells (478), Mononuclear phagocytes (4603), Myocytes (758), Osteoblasts (641), Smooth muscle cells (189) and T cells (329), and the number and UMAP distribution of each cell type were also completely different in the two groups between the control and *K. pintolopesii* treated (**Figures 2A–D**). And the expression of signature markers of each cell type showed as *Gdpd2* and *Csmd1* for MSCs, *Pth1r* and *Coll1a2* for osteoblasts, *Ttn*, *Trdn*, and *Neb* for myocytes, *Cyyr1*, *Ptprb*, and *Pecam1* for ECs, *Ighd*, *Bank1* and *Ighm* for B cells (**Figures 2E, F**), while for the fibroblasts there was no unique gene (**Figures 2E, F**). RO/E (the ratio of observed over expected cell numbers) index suggested that the adipocytes (0.79), B cells (0.21), mononuclear phagocytes (1.17), myocytes (0.67), osteoblasts (0.75), and smooth muscle cells (0.63) might be involved in the process of joint injury induced by *K. pintolopesii* (**Figure 2G**, p < 0.05).

**Figure 3:**
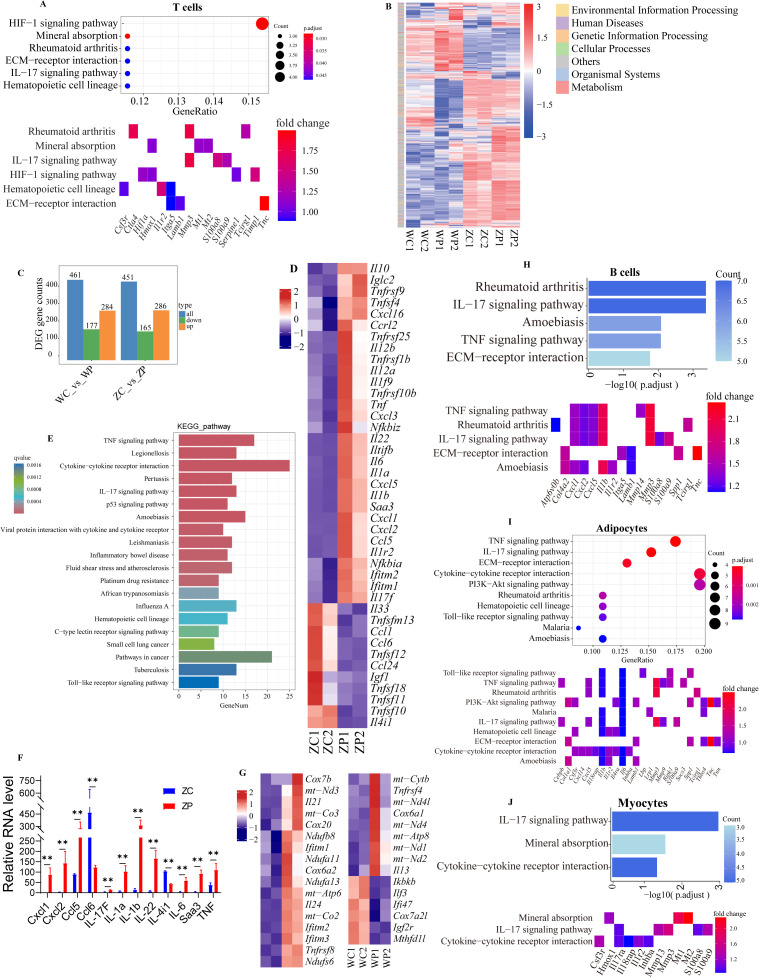
The lysate of *K. pintolopesii* induces severe inflammatory response on the primary spleen T cells of ZAP70^W163C^ mutant mice. KEGG analysis of differentially expressed genes of the T cells from the *K*. *pintolopesii* treated mice using Single-cell Nuclear Sequencing **(A)**; KEGG analysis of differentially expressed genes of the primary spleen T cells using RNA sequencing **(B)**, differentially expressed genes **(C)**, WC indicates the control wild-type mice, WP indicates the *K*. *pintolopesii* treated wild-type mice, ZC indicates the control ZAP70^W163C^ mutant mice, ZP indicates the *K*. *pintolopesii* treated ZAP70^W163C^ mutant mice; KEGG analysis of differentially expressed genes of the primary spleen T cells of *K*. *pintolopesii* treated ZAP70^W163C^ mutant mice using RNA sequencing **(D–G)**; KEGG analysis of differentially expressed genes of the B cells from the *K. pintolopesii* treated mice using single-cell nuclear sequencing **(H)**, Adipocytes **(I)** and Myocytes **(J)**; Data are presented as means ± SD from more than three independent experiments. **p < 0.01 versus the model group, as determined by one-way ANOVA followed by the Holm-Šidák test.

Cell-cell communication through the Interactive CellChat Explorer showed that the interaction networks increased in the *K. pintolopesii* treated group (**Figure 2H**). And the major ligands and receptors of the interaction between T cells and other 9 cell types showed in **Figure 2I**, as *Sema4d*-*Plxnb1*/*Plxnb2*/*Plxnd1*, *Ntn1*-*Unc5b*, *Lgals3*-*Mertk*, Integrin *a4b1*-complex *Plaur* for the ligands of T cells communicated to the receptors of osteoblasts (**Figure 2I**), which indicated that the T cells interact with the receptor protein molecules from the targeted osteoblasts by secreting donor protein molecules or ligands. The top 40 differentially expressed genes showed in **Supplementary Figure S2A**, and the KEGG analysis showed that the pathway of *Hif-1 signaling* (marker genes of *Ctla4*, *Hif1a*, *Serpine1*, *Timp1*), *Rheumatoid arthritis* (*Ctla4*, *Mmp3*, *Tcirg1*), *ECM-receptor interaction* (*Itga5*, *Lamb1*), *IL-17 signaling* (*Mmp3*, *S100a8*, *S100a9*) were influenced (**Figure 3A**), which suggested that *K. pintolopesii* activates these pathways in the T cells of ZAP70^W163C^ mutant mice.

In addition, the lysate of *K. pintolopesii* were added into the primary spleen T cells from ZAP70^W163C^ mutant mice (LKP-TCM). The RNA-sequence of LKP-TCM showed that the inflammatory markers of *Cxcl1*, *IL-22*, *IL-1β*, *Saa3*, *IL-6*, *Cxcl2*, *Ccl5*, *Ccl6*, *Il-4i1*, *IL-1a*, *TNF*, and *IL-17F* were significantly up-regulated (**Figures 3B, D, F**, *p* < 0.05); and the KEGG analysis of up-regulated genes were enriched into *TNF signaling pathway*, *Cytokine-cytokine receptor interaction*, *IL-17 signaling pathway* and *Inflammatory bowel disease* (**Figure 3E**, p *<* 0.05), and these changes may be driven by the genes of *Tcf7*, *Foxp3*, *Batf*, *Gata3* and *Ikzf2* (**Supplementary Figure S2H**), the inflammatory reaction was not so severely while the mitochondrial genes were changed in the LKP-TCM of wildtype mice (**Figure 3G**, p *<* 0.05). All which indicated that the *K. pintolopesii* also acts directly on T cells, then induced severe inflammatory reaction on ZAP70^W163C^ mutant mice, also induced mitochondrial dysfunction in the normal individual.

The changes of other cell type of B cells, adipocytes and myocytes showed in **Figures 3E, F** and **Supplementary Figures S2B–G**, the differentially expressed genes in all the three cell types were enriched in the *IL-17 signaling pathway* (**Figures 3E, F**; **Supplementary Figures S2B–G**). And these changes might be driven by the genes of *Dmrta2*, *Pou2af1*, *Bach2*, *Zfp831* and *Irx5* for B cells; *Srebf1*, *Trp63*, *Thrb*, *Nr1h3* and *Isl1* for adipocytes; *Zfp612*, *Pgam2*, *Esrrg*, *Myod1* and *Pitx2* for myocytes (**Supplementary Figure S2H**). While all the above bioinformatics analysis results need to be verified by test experiments.

### Lysate of *K. pintolopesii* induces severe inflammatory response on the primary medullary macrophage of BALB/c ZAP70^W163C^ mutant mouse

**Figure 4:**
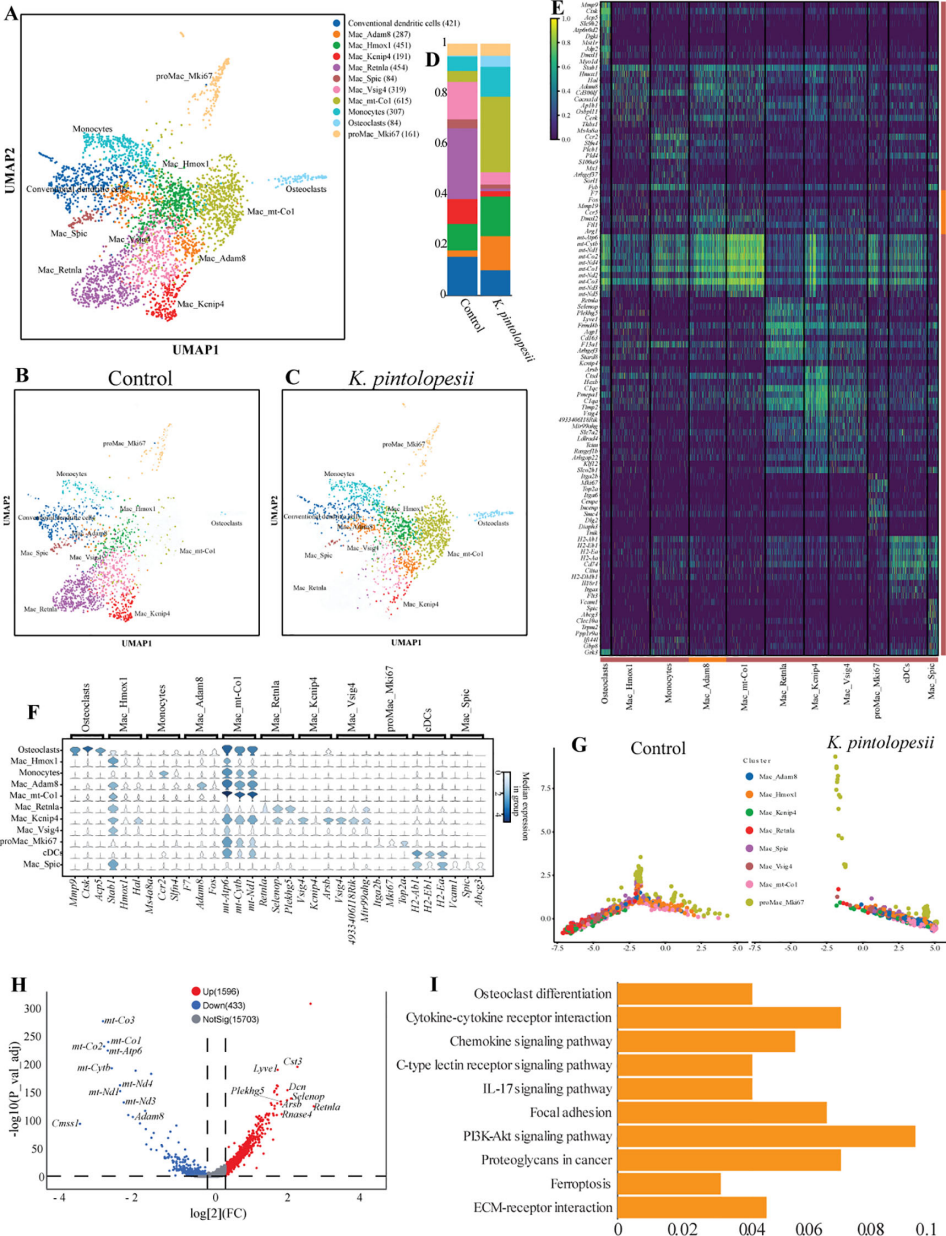
Lysate of *K. pintolopesii* induces severe inflammatory response on the primary medullary macrophage of BALB/c ZAP70^W163C^ mutant mouse. The UMAP visualization illustrates the distribution of eight macrophage subpopulations **(A)**, along with their spatial patterns in both the control **(B)** and *K*. *pintolopesii*-treated groups **(C)**. The proportional abundance of each subpopulation is quantified in **(D)**. Expression of cell-type-specific marker genes for all subpopulations is depicted in **(E, F)**. Pseudotemporal trajectory analysis revealed a completely altered differentiation pattern in the *K*. *pintolopesii*-treated group **(G)**. Differentially expressed genes (DEGs) between *K*. *pintolopesii*-treated and control mice are shown in **(H)**. KEGG pathway enrichment analysis of DEGs from macrophages in the treated group, as identified by single-nucleus RNA sequencing, is presented in **(I)**. Data are presented as means ± SD from more than three independent experiments.

Traditional classifications (e.g., M1/M2 polarization) often oversimplify macrophage heterogeneity by ignoring dynamic functional states, developmental trajectories, and tissue-specific adaptations. To further investigate the functional plasticity, developmental continuity, tissue-specific niches of *K. pintolopesii* on mononuclear phagocytes in BALB/c ZAP70^W163C^ mutant mouse, we performed a new in-depth subpopulation analysis based on preliminary single-nucleus RNA sequencing data. Although initial RO/E index analysis suggested that mononuclear phagocytes were not broadly affected by *K. pintolopesii* (**Figure 2**), further refinement of cell clustering identified 11 distinct subpopulations (**Figure 4A**): conventional dendritic cells (421), Mac_*Adam8* (287), Mac_*Hmox1* (451), Mac_*Kcnip4* (191), Mac_*Retnla* (454), Mac_*Spic* (Hanzelmann et al., 2013), Mac_*Vsig4* (319), Mac_*mt-Co1* (615), ProMac_*Mki67* (161), monocytes (307), and osteoclasts (Hanzelmann et al., 2013). Notably, macrophages were subdivided into eight functionally distinct subsets. Uniform Manifold Approximation and Projection (UMAP) revealed substantially altered distribution patterns in the *K. pintolopesii* treated group compared to controls (**Figures 4B, C**). The relative abundances of most subpopulations were significantly modified following fungal exposure (**Figure 4D**, p < 0.05). Signature markers for each macrophage subset were identified: *Fos* and *F7* for Mac_*Adam8*; *Stab1* and *Hal* for Mac_*Hmox1*; *Vsig4* and *Arsb* for Mac_*Kcnip4*; *Selenop* and *Plekhg5* for Mac_*Retnla*; *Vcam1* and *Abcg3* for Mac_*Spic* and Mac_*Vsig4*; *Itga2b* and *Top2a* for ProMac_*Mki67*; and *mt-Atp6*, *mt-Cytb*, and *mt-Nd1* for Mac_*mt-Co1* (**Figures 4E, F**).

**Figure 5:**
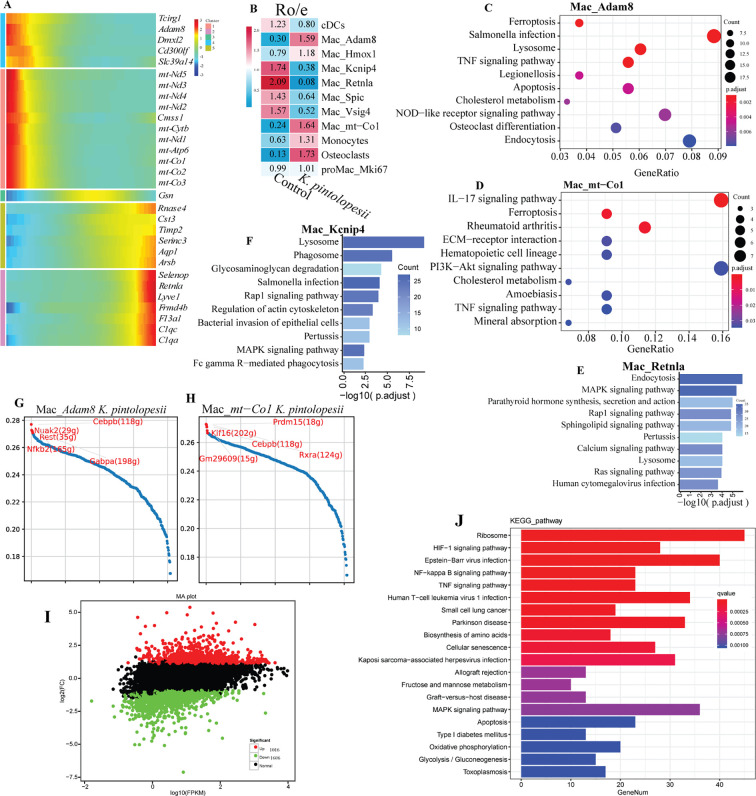
Lysate of *K. pintolopesii* induces severe inflammatory response on the primary medullary macrophage of BALB/c ZAP70^W163C^ mutant mouse. Changes in the expression trends of cell type-specific markers for each macrophage subpopulation **(A)**; RO/E index analysis of the eight macrophage subpopulations **(B)**; KEGG pathway enrichment analysis of differentially expressed genes (DEGs) in macrophage subpopulations from *K*. *pintolopesii* treated mice via single-nucleus RNA sequencing: Mac_*Adam8***(C)**, Mac_*mt-Co1***(D)**, Mac_*Retnla***(E)**, and Mac_*Kcnip4***(F)**; identification of key driver genes for the Mac_*Adam8***(G)** and Mac_*mt-Co1***(H)** subpopulations; DEGs in macrophages from BALB/c ZAP70^W163C^ mutant mice stimulated with *K*. *pintolopesii* lysate **(I)**, and KEGG analysis of these DEGs via RNA sequencing **(J)**. Additional supporting data are provided in **Supplementary Figure S3**. Data are presented as means ± SD from more than three independent experiments.

Pseudotemporal trajectory analysis demonstrated a profound reorganization of cellular states. Cells located in the lower left quadrant in controls either transdifferentiated into other subtypes or were entirely absent in the treated group, indicating a comprehensive reshuffling of macrophage subpopulations induced by *K. pintolopesii* (**Figure 4G**). Differential gene expression analysis between treated and control groups highlighted significant downregulation of mitochondrial genes (**Figure 4H**, see also in **Figure 5A**). KEGG pathway analysis indicated enrichment in *osteoclast differentiation*, *cytokine–cytokine receptor interaction*, *IL-17 signaling* and *PI3K–AKT signaling*, suggesting that *K. pintolopesii* triggers inflammatory responses and mitochondrial oxidative stress in murine hosts (**Figure 4I**). RO/E indices further indicated a positive association of Mac_*Adam8* (1.59) and Mac_*mt-Co1* (1.64) with arthritis and spondylitis, whereas Mac_*Kcnip4* (0.38), Mac_*Retnla* (0.08), Mac_*Spic* (0.64), and Mac_*Vsig4* (0.52) were negatively correlated (**Figure 5B**, p < 0.05). Cell–cell interaction networks revealed enhanced cross-talk among macrophage subgroups in the treated group (**Supplementary Figures S3A, B**). Subpopulation specific KEGG analysis indicated that Mac_*Adam8* exhibited activation of *ferroptosis*, *TNF signaling*, *apoptosis*, *NOD-like receptor signaling*, and *lysosomal pathways*, along with *Salmonella and Legionella infection pathways*, but inhibition of *osteoclast differentiation* and *endocytosis* (**Figure 5C**, p < 0.05). In Mac_*mt-Co1*, *IL-17 signaling*, *ferroptosis*, *TNF signaling*, *rheumatoid arthritis*, and *PI3K–AKT pathways* were significantly activated (**Figure 5D**, p < 0.05). Mac_*Retnla* showed enrichment in *endocytosis*, *MAPK signaling*, and *parathyroid hormone pathways* (**Figure 5E**, p < 0.05), while Mac_*Kcnip4* was enriched in *lysosome and phagosome pathways* (**Figure 5F**, p < 0.05), implying impaired phagocytic function in these subsets upon fungal challenge.

Notably, cholesterol metabolism was upregulated in both Mac_*Adam8* and Mac_*mt-Co1* (**Figures 5C, D**, p < 0.05). Given that aberrant macrophage cholesterol metabolism is known to contribute to rheumatoid arthritis pathogenesis via dysregulated uptake, transport, esterification, and storage, these changes suggest a role in promoting inflammation and bone destruction (Tejera-Segura et al., 2017; Quevedo-Abeledo et al., 2021). Candidate driver genes for Mac_*Adam8* included *Cebpb*, *Nuak2*, *Rest*, *Nfkb2*, and *Gabpa* (**Figure 5G**, p < 0.05), while *Trp63*, *Gm35315*, *Six1*, *Hoxa2*, and *Zfp3*were identified as potential therapeutic targets. For Mac_*mt-Co1*, central regulators included *Prdm15*, *Klf16*, *Cebpb*, *Gm29609*, and *Rxra* (**Figure 5H**, p < 0.05), with *Bnc2*, *Plagl1*, *Sp7*, *Churc1*, and *Gm14399*representing possible targets for intervention (**Supplementary Figure S3C**, p < 0.05).

To validate these findings, bone marrow-derived macrophages from BALB/c ZAP70^W163C^ mutant mice (BMDM-ZAP70) were treated with *K. pintolopesii* lysate (LKP). RNA sequencing identified 2,622 differentially expressed mRNAs (**Figure 5I**, p < 0.05; **Supplementary Figure S4A**, *p* < 0.05). Proinflammatory cytokines (*IL-1β*, *IL-6*, *IL-7*, *IL-18*, *TNF-α*) were markedly upregulated (**Supplementary Figures S4A–D**, *p* < 0.05), along with PANoptosome components (*ZBP1*, *RIPK3*, *RIPK1*, *caspase-8*, *caspase-6*, *ASC*, *NLRP3*) and interferon-related genes (**Supplementary Figures S4A, B**, *p* < 0.05). KEGG analysis of upregulated genes indicated activation of *HIF-1*, *NF-κB*, and *TNF signaling pathways* (**Figure 5J**, p < 0.05), suggesting integrated dysregulation of oxygen homeostasis, immune activation, and energy metabolism that sustains chronic inflammation and tissue remodeling. Enrichment of pathways related to *Epstein-Barr virus*, *human T-cell leukemia virus 1*, and *Kaposi sarcoma-associated herpesvirus infections* implied broad compromise of immune defense mechanisms (**Figure 5J**, p < 0.05). Conversely, downregulated genes were enriched in pathways including *herpes simplex virus 1 infection*, *NOD-like receptor signaling*, and *apoptosis* (**Supplementary Figure S3D**, *p* < 0.05). Collectively, these results demonstrate that LKP induces severe inflammatory responses in BMDM-ZAP70 macrophages.

### Lysate of *K. pintolopesii* induce osteoblast fibrosis of BALB/c ZAP70^W163C^ mutant mouse

**Figure 6:**
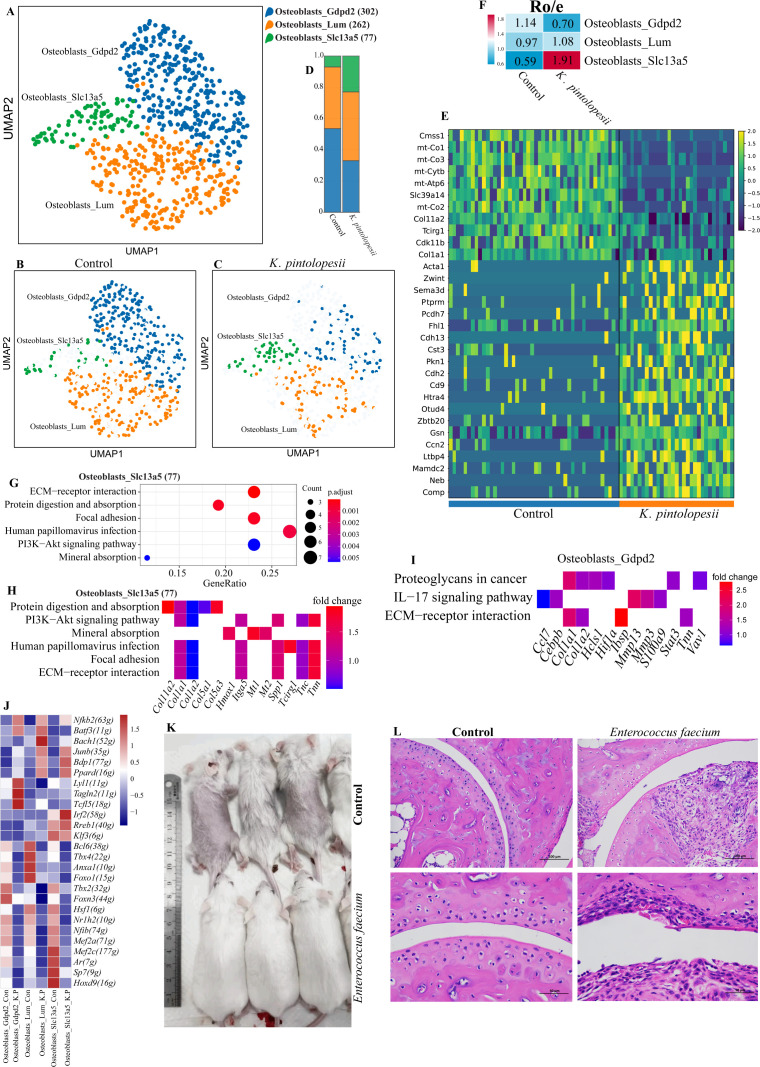
Lysate of *K. pintolopesii* induce osteoblast fibrosis of BALB/c ZAP70^W163C^ mutant mouse. The UMAP visualization illustrates the distribution of osteoblast subpopulations **(A)**, along with their distinct spatial patterns in both the control **(B)** and *K*. *pintolopesii*-treated groups **(C)**. The proportional abundance of each osteoblast subpopulation is quantified in **(D)**. Expression of cell-type-specific marker genes for the osteoblast subpopulations is depicted in **(E)**. The ratio of observed to expected (RO/E) index was calculated to evaluate the enrichment or depletion of each osteoblast subpopulation across conditions **(F)**. KEGG pathway enrichment analysis of differentially expressed genes (DEGs) in osteoblast subpopulations from *K*. *pintolopesii*-treated mice, assessed via single-nucleus RNA sequencing, is presented for osteoblasts_*Slc13a5***(G, H)** and osteoblasts_*Gapd2***(I)**. A heatmap displays the differential expression of transcription factors across each osteoblast subpopulation **(J)**. Fermentation products of *Enterococcus faecium* were observed to ameliorate or even reverse pathological symptoms such as hair loss and joint swelling **(K, L)**; further supporting data are provided in **Supplementary Figure S5**. Data are presented as means ± SD from more than three independent experiments.

Further analysis revealed that osteoblasts could be subdivided into three distinct subpopulations (**Figure 6A**). Both UMAP visualization and cell proportion analyses demonstrated significant alterations following *K. pintolopesii* treatments (**Figures 6B–D**, p < 0.05). In the osteoblasts_*Slc13a5* subpopulation, signature marker genes including *Cmss1*, *mt-Co1*, *mt-Co3*, *mt-Cytb*, *mt-Atp6*, *Slc39a14*, *mt-Co2*, *Col11a2*, *Tcirg1*, *Cdk11b*, and *Col1a1*were significantly downregulated (**Figure 6E**, p < 0.05), while transcription factors such as *Junb*, *Bdp1*, *Ppard*, *Irf2*, *Rreb1*, and *Klf3* were upregulated (**Figure 6H**, p < 0.05).

The RO/E index indicated that the osteoblasts_*Gapd2* subpopulation (0.70) was negatively associated with arthritis and spondylitis, whereas osteoblasts_*Slc13a5* (1.91) showed a positive correlation (**Figure 6F**, p < 0.05). KEGG enrichment analysis of differentially expressed genes highlighted several key pathways, including *ECM-receptor interaction* (involving *Tnn*, *Tnc*, *Spp1*, *Itga5*, *Col1a1*, and *Col1a2*), *protein digestion and absorption* (*Col11a2*, *Col1a1*, *Col1a2*, *Col5a1*, and *Col5a3*), and the *PI3K-Akt signaling pathway* (*Col1a1*, *Col1a2*, *Tnn*, *Tnc*, *Spp1*, and *Itga5*) (**Figures 6G, H**, p < 0.05). In the osteoblasts_*Gapd2* subpopulation, pathways such as *proteoglycans in cancer* (*Col1a1*, *Col1a2*, *Hcls1*, *Hif1a*, *Stat3*, *Vav1*), *IL-17 signaling* (*Mmp13*, *Mmp3*, *S100a9*, *Ccl7*, *Cebpb*), and *ECM–receptor interaction* (*Col11a2*, *Col1a1*, *Ibsp*, and *Tnn*) were activated (**Figure 6I**, p < 0.05). Transcription factors including *Junb*, *Bdp1*, *Lyl1*, *Tagln2*, *Tcfl5*, *Tbx2*, and *Foxn3*may contribute to these changes (**Figure 6J**, p < 0.05). Additionally, fermentation products of *Enterococcus faecium* not only alleviated but also reversed pathological symptoms—including alopecia and joint swelling—with statistical significance (**Figures 6K, L**, p < 0.05). Further details regarding the therapeutic efficacy and underlying mechanisms of *E. faecium* will be elucidated in our subsequent manuscript.

### Lysate of *K. pintolopesii* promote the fibroblast-like synoviocyte abnormal proliferation

**Figure 7:**
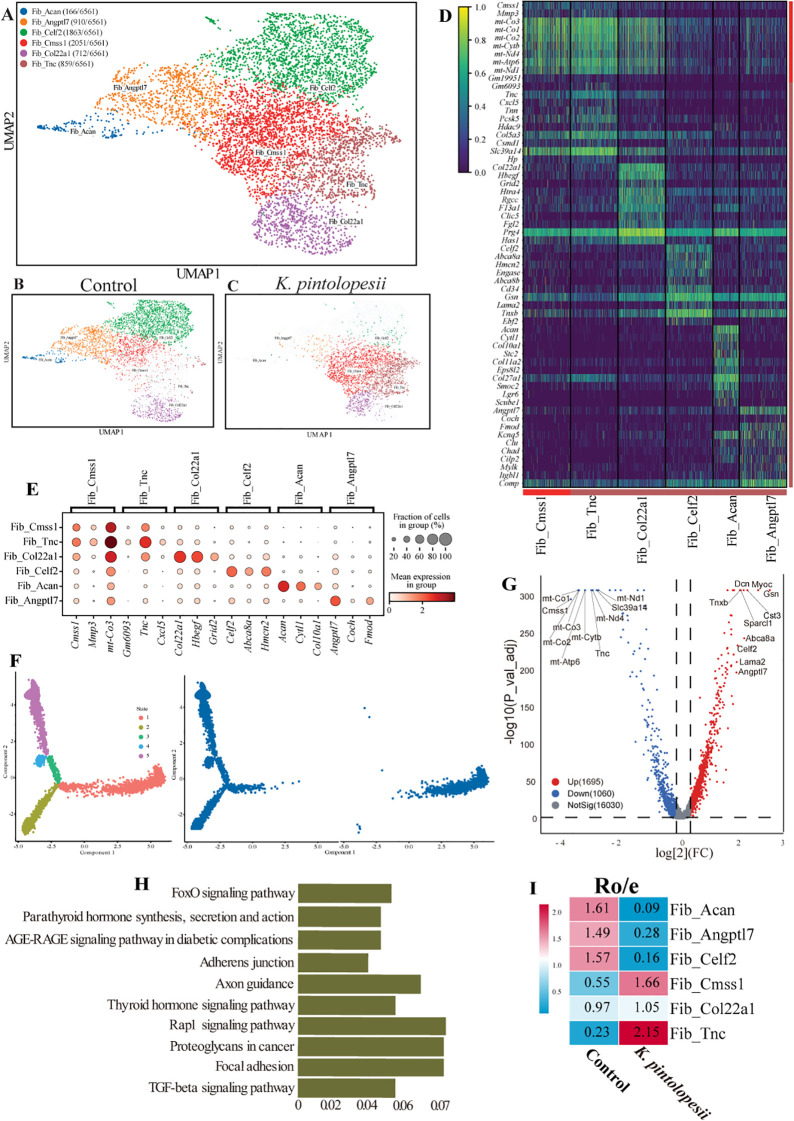
Lysate of *K. pintolopesii* promote the fibroblastlike synoviocyte abnormal proliferation of BALB/c ZAP70^W163C^ mutant mouse. The UMAP visualization illustrates the distribution of fibroblast-like synoviocyte (FLS) subpopulations **(A)**, along with their spatial patterns in both the control **(B)** and *K*. *pintolopesii*-treated groups **(C)**. Expression of cell-type-specific marker genes for each FLS subpopulation is depicted in **(D, E)**. Pseudotemporal trajectory analysis revealed a completely altered differentiation pattern in the *K*. *pintolopesii*-treated group **(F)**. Differentially expressed genes (DEGs) between *K*. *pintolopesii*-treated and control mice are shown in **(G)**. KEGG pathway enrichment analysis of DEGs from macrophages in the treated group, as identified by single-nucleus RNA sequencing, is presented in **(H)**. The RO/E index was calculated to evaluate the enrichment or depletion of each FLS subpopulation across conditions **(I)**. Data are presented as means ± SD from more than three independent experiments. *p < 0.05 and **p < 0.01 versus the model group, as determined by one-way ANOVA followed by the Holm-Šidák test.

Following treatment with *K. pintolopesii*, fibroblast-like synoviocytes (FLS) were subdivided into six distinct subpopulations (**Figure 7A**), Fib_*Acan* (166), Fib_*Angptl7* (910), Fib_*Celf2* (1863), Fib_*Cmss1* (2051), Fib_*Col22a1* (712), and Fib_*Tnc* (859). UMAP analysis revealed marked alterations in the spatial distribution of these subpopulations in the treated group (**Figures 7B, C**, p < 0.05). Signature marker expression for each subpopulation is detailed in **Figures 7D, E**. Pseudotemporal trajectory analysis indicated a substantial disruption in the developmental path of FLS following fungal exposure (**Figure 7F**, p < 0.05). Differential gene expression analysis identified 1695 upregulated and 1060 downregulated genes in the *K. pintolopesii* treated group (**Figure 7G**, p < 0.05). Notably, the most significantly downregulated genes were mitochondrial, including *mt-Co1*, *mt-Co3*, *mt-Atp6*, *mt-Cytb*, *mt-Nd1*, and *mt-Nd4* (**Figure 7G**, p < 0.05). Pathway enrichment analysis revealed activation of *FoxO signaling*, *axon guidance*, *Rap1 signaling*, *focal adhesion*, and *TGF-β signaling pathways* (**Figure 7H**, p < 0.05).

**Figure 8:**
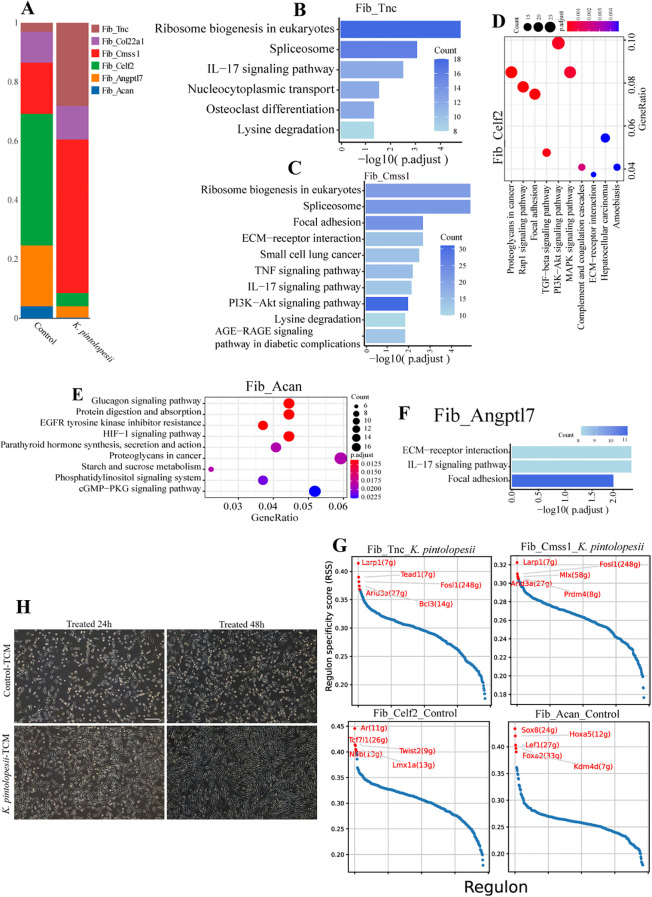
Lysate of *K. pintolopesii* promote the fibroblastlike synoviocyte abnormal proliferation of BALB/c ZAP70^W163C^ mutant mouse. The proportion of each fibroblastlike synoviocyte subpopulations **(A)**; KEGG analysis of differentially expressed genes of the fibroblastlike synoviocyte subpopulations of the *K*. *pintolopesii* treated mice using single-cell nuclear sequencing, Fib_*Tnc***(B)**, Fib_*Cmss1***(C)**, Fib_*Celf2***(D)**, Fib_*Acan***(E)**, and Fib_*Angptl7***(F)**; RSS analysis of Fib_*Tnc*, Fib_*Cmss1*, Fib_*Celf2* and Fib_*Acan***(G)**, and more details showed in **Supplementary Figure S6**. Conditioned medium of primary spleen T cells from ZAP70^W163C^ mutant mice treated by LKP induced rapid proliferation and fibrosis of muscle stem cells. Data are presented as means ± SD from more than three independent experiments. *p < 0.05 and **p < 0.01 versus the model group, as determined by one-way ANOVA followed by the Holm-Šidák test.

The RO/E index indicated that Fib_*Acan* (0.09), Fib_*Angptl7* (0.28), and Fib_Celf2 (0.16) were negatively correlated with arthritis and spondylitis, whereas Fib_*Cmss1* (1.66) and Fib_*Tnc* (2.15) showed positive correlations (**Figure 7I**, p < 0.05). The relative abundance of most subpopulations was significantly altered post-treatment (**Figure 8A**, p < 0.05). In particular, a substantial proportion of cells differentiated into the Fib_*Cmss1* (marked by *Cmss1*, *Mmp3*, and *mt-Co3*; **Figures 7D, E**) and Fib_*Tnc* (marked by *Tnc*, *Cxcl5*, and *Gm6093*; **Figures 7D, E**) subsets, suggesting that these two subpopulations may contribute to the loss of clear tissue and organ boundaries observed in **Figures 1C** and **6J, K**.

Further pathway analysis in the Fib_*Tnc* subpopulation showed significant enrichment in Ribosome biogenesis in *eukaryotes* (key genes: *Acin1*, *Ddx39b*, *Rbm25*, *Stf3b1*; **Supplementary Figure S5A**), *Spliceosome* (*Acin1*, *Ddx39b*, *Rbm25*, *Stf3b1*), IL-17 signaling (*Cxcl5*, *IL-17RA*, *IL-1b*, *IL-1R1*, *IL-6*, *Mmp13*, *S100a9*), *Nucleocytoplasmic transport* (*Acin1*, *Ddx39b*, *IL-6*, *Nv1*), *Osteoclast differentiation* (*Ncf2*, *Fosl1/2*, *IL-1b*, *Snrnp70*), *Lysine degradation* (*Cebpb*, *Ipo5*, *Sart1*, *Sf3b3*) (**Figure 8B**, p < 0.05). Additionally, the Fib_*Cmss1* subpopulation exhibited activation of *focal adhesion*, *TNF signaling*, *IL-17 signaling*, and *PI3K-AKT signaling pathways* (**Figure 8C**, p < 0.05; **Supplementary Figure S5B**). The Fib_*Celf2* subpopulation showed enrichment in *Rap1 signaling*, *TGF-β signaling*, *MAPK signaling*, and *PI3K-AKT pathways* (**Figure 8D**, p < 0.05; **Supplementary Figure S5C**). Fib_*Acan* was associated with *glucagon signaling*, *protein digestion and absorption*, *HIF-1 signaling*, and *cGMP-PKG signaling pathways* (**Figure 8E**, p < 0.05; **Supplementary Figure S5D**), while Fib_*Angptl7* demonstrated activation of *ECM-receptor interaction*, *IL-17 signaling*, and *focal adhesion pathways* (**Figure 8F**, p < 0.05; **Supplementary Figure S5E**). These findings collectively indicate an expansion of pro-inflammatory fibroblast subpopulations in *K. pintolopesii* treated mice. RSS (Regulon specificity score) analysis identified potential driver genes for each subpopulation, *Larp1*, *Tead1*, *Fosl1*, *Arid3a*, and *Bcl3* for Fib_*Tnc*; *Larp1*, *Mlx*, *Arid3a*, *Fosl1*, and *Prdm4* for Fib_*Cmss1*; *Ar*, *Tcf7l1*, *Twist*, *Nfib*, and *Lmx1a* for Fib_*Celf2*; and *Sox8*, *Hoxa5*, *Lef1*, *Foxa2*, and *Kdm4d* for Fib_*Acan* (**Figure 8G**, p < 0.05).

In a complementary experiment, primary spleen T cells from ZAP70^W163C^ mutant mice were treated with *K. pintolopesii* lysate (LKP), and the resulting conditioned medium was applied to muscle stem cells (MuSCs) in proliferation medium. This treatment induced rapid proliferation and fibrosis of MuSCs (**Figure 8H**). However, when the same conditioned medium was applied to differentiation medium, no obvious fibrotic or differentiation effects were observed (data not shown). These results suggest that *K. pintolopesii* may initiate fibrosis in muscle and/or bone tissues by activating stem cells (such as muscle stem cells or periosteal stem cells), though further validation is required to substantiate this mechanism.

### Lysate of *K. pintolopesii* influence the endothelial cells significant changed

In rheumatoid arthritis (RA), endothelial cells (ECs) are now recognized as active contributors to disease pathogenesis rather than passive targets. They promote disease progression by regulating central inflammatory processes such as leukocyte extravasation, angiogenesis, cytokine release, and protease production (Nygaard and Firestein, 2020; Wei et al., 2020; Zhao et al., 2023). Endothelial dysfunction compromises vascular integrity, facilitates leukocyte infiltration, and exacerbates synovial hypoxia, thereby accelerating synovial hyperplasia and joint damage (Patidar et al., 2022; Ahmed et al., 2023).

**Figure 9:**
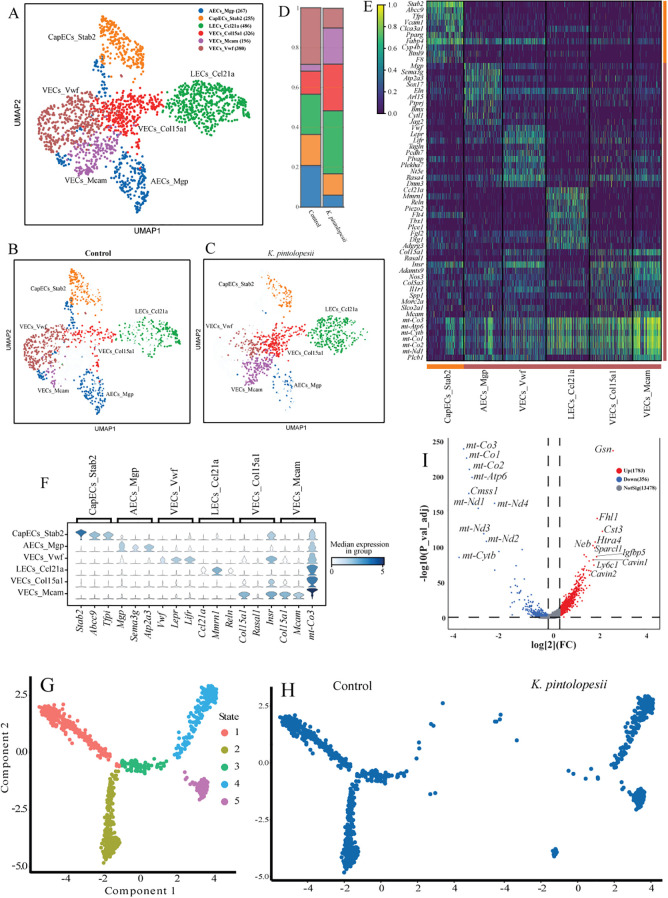
Lysate of *K. pintolopesii* influence the endothelial cells significant changed on BALB/c ZAP70^W163C^ mutant mouse. The UMAP visualization illustrates the distribution of endothelial cell subpopulations **(A)**, along with their spatial patterns in both the control **(B)** and *K*. *pintolopesii*-treated groups **(C)**. The proportional abundance of each endothelial subpopulation is quantified in **(D)**. Expression of cell-type-specific marker genes for all endothelial subpopulations is depicted in **(E, F)**. Pseudotemporal trajectory analysis revealed a markedly altered differentiation pattern in the *K*. *pintolopesii*-treated group **(G, H)**. Differentially expressed genes (DEGs) between the *K*. *pintolopesii*-treated and control mice are shown in **(I)**. Data are presented as means ± SD from more than three independent experiments. *p < 0.05 and **p < 0.01 versus the model group, as determined by one-way ANOVA followed by the Holm-Šidák test.

In this study, we further dissected the heterogeneity of synovial ECs and identified six distinct subpopulations (**Figure 9A**), AECs_*Mgp* (267), CapECs_*Stab2* (255), LECs_*Ccl21a* (486), VECs_Col15a1 (326), VECs_*Mcam* (196), and VECs_*Vwf* (380). UMAP revealed pronounced compositional shifts in the *K. pintolopesii* treated group relative to controls (**Figures 9B, C**), with significant alterations in the abundance of most subpopulations (**Figure 9D**, p < 0.05). Key marker genes for each subset were identified, CapECs_*Stab2*: *Stab2*, *Abcc9*, *Tfpi*; AECs_*Mgp*: *Mgp*, *Sema3g*, *Atp2a3*; LECs_*Ccl21a*: *Ccl21a*, *Mmmrn1*, *Reln*; VECs_*Col15a1*: *Col15a1*, *Rasal1*, *Insr*; VECs_*Mcam*: *Col15a1*, *Mcam*, *mt-Co3*; VECs_*Vwf*: *Vwf*, *Lepr*, *Lifr* (**Figures 9E, F**). Pseudotemporal trajectory analysis demonstrated a substantial reorganization of cellular states. Clusters corresponding to states 1–3 in controls were entirely absent or transformed into other subtypes following *K. pintolopesii* exposure (**Figures 9G, H**), indicating profound disruption of endothelial subpopulation architecture.

**Figure 10:**
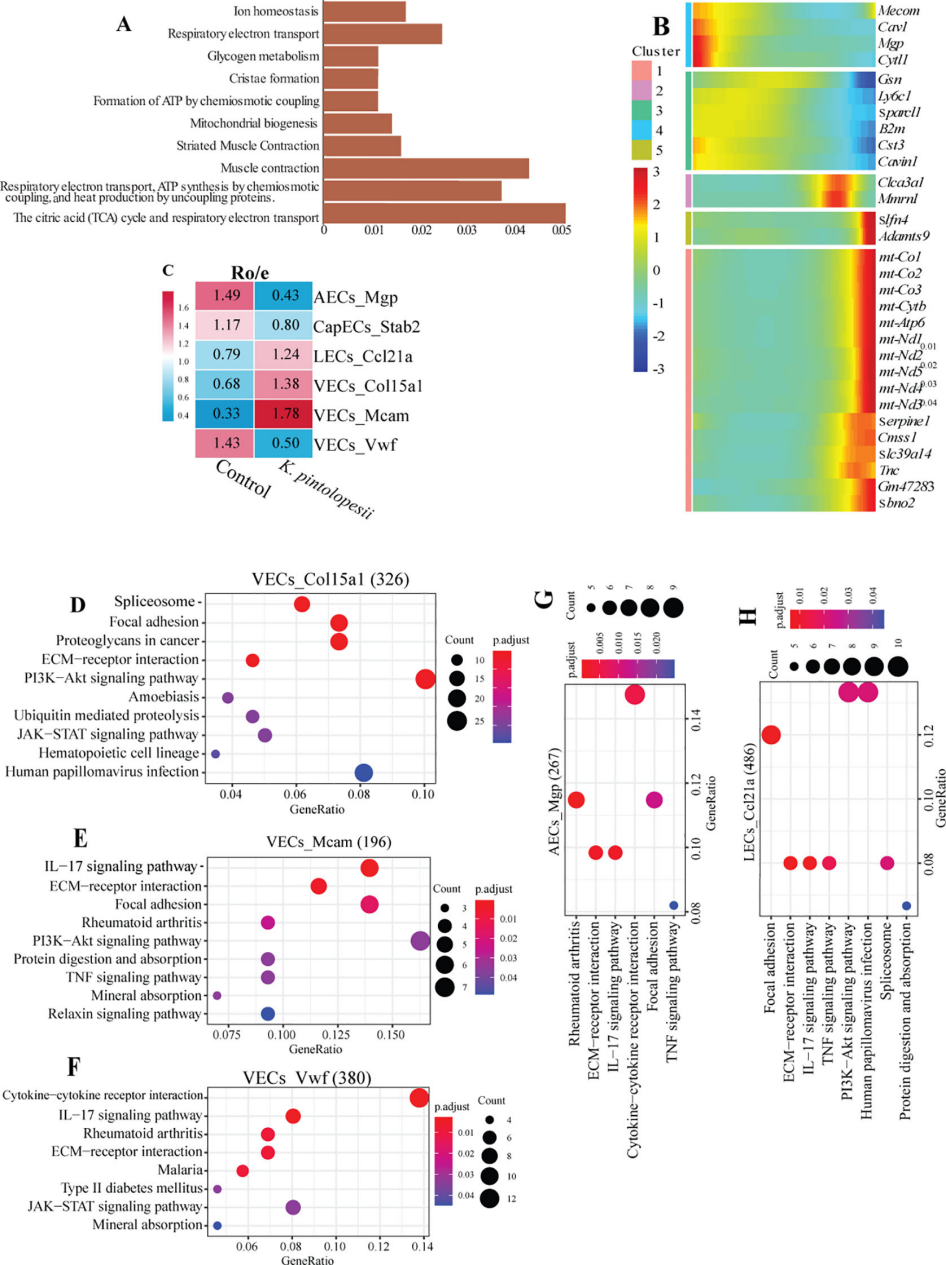
Lysate of *K. pintolopesii* influence the endothelial cells significant changed on BALB/c ZAP70^W163C^ mutant mouse. KEGG pathway analysis was performed on differentially expressed genes (DEGs) from endothelial cells of *K*. *pintolopesii*-treated mice using single-nucleus RNA sequencing data **(A)**. The expression trends of cell-type-specific markers for each endothelial subpopulation are displayed in **(B)**. The ratio of observed to expected (RO/E) index for evaluating the enrichment or depletion of endothelial subpopulations is shown in **(C)**. KEGG analysis of DEGs was conducted for the following endothelial subpopulations from *K*. *pintolopesii*-treated mice using single-nucleus RNA sequencing: VECs_*Col15a1***(D)**, VECs_*Mcam***(E)**, VECs_*Vwf***(F)**, AECs_*Mgp***(G)**, and LECs_*Ccl21a***(H)**. Additional supporting details are provided in **Supplementary Figure S7**. Data are presented as means ± SD from more than three independent experiments. *p < 0.05 and **p < 0.01 versus the model group, as determined by one-way ANOVA followed by the Holm-Šidák test.

Notably, mitochondrial genes (e.g., *mt-Co3*, *mt-Co2*, *mt-Co1*, *mt-Atp6*, *Cmss1*, *mt-Nd1*, *mt-Nd4*, *mt-Nd3*, *mt-Nd2*, *mt-Cytb*) were significantly downregulated in the treated group, whereas genes including *Gsn*, *Fhl1*, *Cst3*, *Htra4*, *Neb*, *Sparcl1*, *Igfbp5*, *Cavin1*, and *Cavin2*were upregulated (**Figure 9I**, p < 0.05). KEGG pathway analysis suggested that these changes reflect altered mitochondrial function, impaired migratory capacity, dysregulated stress responses, and modifications in *IGF signaling*, *cell adhesion*, and *proliferation-related pathways* (**Figure 10A**), and **Figure 10B** details expression trends of marker genes across all six subpopulations.

The RO/E index indicated that VECs_*Mcam* (1.78) and VECs_*Col15a1* (1.38) were positively correlated with arthritis and spondylitis, whereas AECs_*Mgp* (0.43) and VECs_*Vwf* (0.50) were negatively correlated (**Figure 10C**, p < 0.05). In VECs_*Mcam* and VECs_*Col15a1* subpopulations that expanded significantly after treatment (**Figures 9B–D**), KEGG analysis revealed activation of *focal adhesion*, *ECM-receptor interaction*, and *PI3K-Akt signaling pathways* (**Figures 10D, E**, p < 0.05). Additionally, VECs_*Mcam* exhibited enrichment in *rheumatoid arthritis*, *IL-17 signaling*, and *TNF signaling pathways* (**Figure 10E**, p < 0.05; see also **Supplementary Figure S6**), suggesting that VECs_*Col15a1* may be involved in stress sensing and transduction, while VECs_*Mcam* likely contributes to inflammatory initiation. Conversely, AECs_*Mgp* and VECs_*Vwf* showed activation of *cytokine-cytokine receptor interaction*, *ECM-receptor interaction*, and *IL-17 signaling* (**Figures 10F, G**, p < 0.05; **Supplementary Figure S6**), implying that these subpopulations may respond to cytokine signals from VECs_*Mcam* and VECs_*Col15a1*, thereby amplifying stress and inflammatory responses.

## Discussion

The mechanism by which fungal infections contribute to rheumatoid diseases is not fully understood but may involve several factors. One possible mechanism is molecular mimicry, where fungal antigens share structural similarities with self-antigens, leading to immune activation and autoantibody production (Lin et al., 2016). For example, some fungi produce proteins resembling human cartilage proteins, which may trigger an immune response against joint tissues (Geyer et al., 1999; Mostafavi et al., 2022). Another mechanism is the induction of inflammation: fungal infections can cause the release of pro-inflammatory cytokines and chemokines, attracting immune cells to the site of infection and promoting chronic inflammation, a key feature of rheumatoid diseases (Delliere et al., 2024; Zhang et al., 2024; Omaru et al., 2025). Additionally, fungal infections may indirectly contribute to rheumatoid diseases by altering the gut microbiota (Bedoya et al., 2013). The gut microbiota plays a crucial role in immune regulation, and dysbiosis has been linked to autoimmune diseases (Jensen et al., 2024; Bhunyakarnjanarat et al., 2025). Fungal infections can disrupt the balance of the gut microbiota, leading to increased inflammation (Auchtung et al., 2022; Bhunyakarnjanarat et al., 2025).

Some studies have reported an increased prevalence of fungal infections in patients with rheumatoid diseases compared to healthy controls. For example, patients with rheumatoid arthritis show higher colonization of *Candida albicans* in the oral cavity and gut and an increased risk of invasive fungal infections (Bishu et al., 2014; Vaquero-Herrero et al., 2020; Xing et al., 2024). Animal models have also been used to study the role of fungal infections in rheumatoid diseases (Abou-El-Naga et al., 2020); some studies have shown that fungal infections can induce arthritis in mice (Gamaletsou et al., 2022; Kong and Kong, 2025), suggesting a potential role in pathogenesis. Despite growing evidence, the exact relationship remains unclear, and more research is needed to determine the specific mechanisms and develop targeted therapies. In conclusion, microbial infections, particularly fungal infections, may play a significant role in the pathogenesis of rheumatoid diseases. Further research is essential to understand the complex interactions between fungi and the immune system and to develop novel therapeutic strategies.

*Kazachstania pintolopesii* is a yeast species that has garnered increasing attention in recent years. It belongs to the family Saccharomycetaceae and has been isolated from diverse sources including soil, water, plants, and insects (Peng et al., 2021; James et al., 2022). Additionally, it is present in certain fermented foods and beverages. *K. pintolopesii* exhibits several distinctive physiological traits, it can grow across a broad range of temperatures (10-37°C) and pH values (3.0-8.0), tolerate high concentrations of salt and sugar, and produce various enzymes and metabolites such as amylase, protease and ethanol (Peng et al., 2021; James et al., 2022).

This study elucidates the intricate mechanisms through which *K. pintolopesii* lysate (LKP) induces severe inflammatory responses and autoimmune pathology in BALB/c ZAP70^W163C^ mutant mice. Our findings reveal a multifaceted interplay among fungal components, immune dysregulation, and metabolic reprogramming that collectively drive chronic inflammation and tissue destruction. Direct injection of LKP into spinal tissues induced pronounced articular and spinal pathologies, including ulceration, joint swelling, and deformity (**Figure 1**). Histopathological analysis revealed synovial hyperplasia, inflammatory cell infiltration, and cartilage destruction, accompanied by elevated levels of pro-inflammatory cytokines (IL-1β, IL-6, NF-κB) in spinal and joint tissues (**Figure 1**; **Supplementary Figure S1**). These observations align with previous studies linking fungal dysbiosis to autoimmune responses. For example, *K. pintolopesii* has been shown to activate IL-17RA signaling in intestinal tissues, promoting neutrophil recruitment and epithelial barrier disruption (Zhang et al., 2022). Here, we extend this paradigm to the musculoskeletal system, suggesting that fungal components may act as pathobionts that breach immune tolerance and initiate cross-organ inflammation, impacting multiple cell types (**Figures 2**-**10**).

T cells play a pivotal role in the pathogenesis of ankylosing spondylitis (AS). CD4+ T cells, particularly Th17 cells, are increased in the peripheral blood and synovial fluid of AS patients (Su et al., 2022; Akhter et al., 2023). Th17 cells produce cytokines such as IL-17, IL-22, and TNF-α, which promote inflammation and bone erosion. Additionally, regulatory T cells (Tregs) may be dysfunctional in AS, leading to an imbalance between pro-inflammatory and anti-inflammatory T cells (McGinty et al., 2020; Yi et al., 2023). In this study, T cells exhibited a robust inflammatory response to *K. pintolopesii* both *in vivo* (**Figure 2**) and *in vitro* (**Figure 3**). The dysregulated IL-17/TNF axis suggests a shift toward pro-inflammatory Th17 responses, potentially exacerbating autoimmune pathology. Interaction networks between T cells and osteoblasts, fibroblasts, and endothelial cells (**Figures 2H**, **8H**) further underscore the complexity of immune-matrix crosstalk in disease progression. T cells secreted ligands (e.g., *Sema4d*, *Lgals3*) that bound to osteoblast receptors (*Plxnb1*, *Mertk*), promoting osteoclast activation-a process critical in bone remodeling during AS.

B cells may also contribute to AS pathogenesis. Some studies have reported increased levels of autoantibodies (e.g., anti-keratin and anti-collagen antibodies) in AS patients (Soltani et al., 2024; Xie et al., 2025). B cells can produce cytokines and immunoglobulins that activate immune responses and promote inflammation (Deng et al., 2024; Mahyari et al., 2025). The RO/E index indicated that B cells decreased from 1.56 in the control group to 0.21 in the *K. pintolopesii* treated group (**Figure 2G**, p < 0.05). Macrophage analysis identified Mac_*Adam8* and Mac_*mt-Co1* subpopulations as key drivers of osteoclastogenesis and fibrosis, respectively; these subtypes exhibited activated *ferroptosis* and *PI3K-AKT pathways*, correlating with bone erosion and cartilage degradation (**Figure 5**). Altered endothelial cell populations (VECs_*Mcam* and VECs_*Col15a1*) showed disrupted *ECM-receptor interactions* and *IGF signaling*, suggesting impaired vascular homeostasis and leukocyte extravasation (**Figure 9**). Study have proved that macrophage depletion significantly increased the increased fecal Ascomycota, especially *K. pintolopesii*, with polymicrobialbacteremia (*Klebsiella pneumoniae*, *Enterococcus faecalis*, and *Acinetobacter radioresistens*), and increased the mortality and severity of sepsis-CLP mice (Hiengrach et al., 2023), suggesting that the fecal fungus could be spontaneously elevated and altered in response to macrophage-depleted therapy. That is to say, similarly, macrophages also play a very important role in resisting *K. pintolopesii* bacterial infection.

The interplay among *K. pintolopesii*, endothelial cells (ECs), and fibroblast-like synoviocytes (FLS) constitutes a critical pathway in the pathogenesis of inflammatory arthritis and tissue fibrosis. Single-nucleus sequencing data revealed significant alterations in EC subpopulations and FLS phenotypes following LKP treatment, indicating a coordinated response that promotes a pro-fibrotic microenvironment. LKP treatment upregulated the subpopulations of VECs_*Mcam* and VECs_*Col15a1*, which exhibited enriched pathways in *focal adhesion*, *ECM-receptor interaction*, and *PI3K-Akt signaling* (**Figures 10D, E**). These activated ECs secrete proinflammatory cytokines (e.g., IL-1β, TNF-α) and matrix metalloproteinases (MMPs), disrupting vascular integrity and facilitating leukocyte extravasation. Activation of *IL-17* and *TNF signaling pathways* in VECs_*Mcam* (**Figure 10E**) aligns with studies showing that endothelial IL-17R signaling promotes neutrophil recruitment and angiogenesis in rheumatoid synovium (Kehlen et al., 2002). A hallmark of LKP-induced endothelial dysfunction was the significant downregulation of mitochondrial genes (e.g., *mt-Co1*, *mt-Atp6*) and upregulation of stress-response genes (e.g., *Gsn*, *Fhl1*) (**Figure 9I**). This metabolic shift toward glycolysis (evidenced by HIF-1α pathway activation) amplifies inflammatory responses and enhances lactate production, which can act as a paracrine signal to activate fibroblasts, inducing collagen synthesis and promoting fibrosis. The emergence of pro-fibrotic FLS subpopulations (Fib_*Cmss1* and Fib_*Tnc*) with enriched *ECM-receptor interaction*, *PI3K-AKT*, and *TGF-β signaling pathways* (**Figures 8C–F**) underscores the role of fungal components in driving synovial fibrosis. These fibroblasts likely respond to endothelial-derived factors (e.g., *TGF-β1*, *PDGF*) and matrix cues (e.g., stiffened ECM), undergoing phenotypic transformation into myofibroblasts that secrete excessive collagen and perpetuate tissue scarring. Upregulation of Tnc (tenascin-C) in Fib_*Tnc* is particularly notable, as it promotes fibroblast migration and adhesion in fibrotic niches.

Although our data did not directly assay IL-33 in synovial tissues, recent studies indicate that *K. pintolopesii* can induce epithelial IL-33 expression in intestinal mucosa, triggering ST2-dependent type 2 immunity (Liao et al., 2024). In the context of joint inflammation, IL-33 released from stressed endothelial or epithelial cells could activate group 2 innate lymphoid cells (ILC2s) and mast cells, fostering a pro-fibrotic milieu through Th2 cytokines (IL-4, IL-13) and TGF-β1. This pathway warrants further investigation in musculoskeletal fibrosis (Sheets et al., 2022). Cytokines are key mediators of inflammation in AS. TNF-α, IL-17, IL-23, and other cytokines are elevated in the serum and synovial fluid of AS patients (Ritchlin and Adamopoulos, 2021; Lems et al., 2022). These cytokines promote the recruitment and activation of immune cells, leading to inflammation and bone erosion (Sode et al., 2018; Venetsanopoulou et al., 2020). In this study, single-nucleus sequencing revealed dramatic shifts in immune cell populations and demonstrated that *K. pintolopesii* remodels the immune cell network and increases the complexity of cell-cell crosstalk. Notably, in T cells, IL-17A/F, TNF-α, and CXCL1/2 were upregulated in LKP-treated mice, with KEGG enrichment of *IL-17* and *TNF signaling pathways* (**Figure 3**), consistent with findings in rheumatoid arthritis where IL-17-producing Th17 cells drive synovial inflammation.

A striking feature of our findings is the downregulation of mitochondrial genes (e.g., *mt-Co1*, *mt-Atp6*) in osteoblasts, macrophages, and endothelial cells (**Figures 6E**, **9I**). This mitochondrial dysfunction likely contributes to oxidative stress and energy deficits, perpetuating inflammatory cascades. Previous studies have linked mitochondrial stress to NLRP3 inflammasome activation and pyroptosis (Zhang et al., 2023; Pandey et al., 2025), a mechanism implicated in *K. pintolopesii* induced PANoptosis in macrophages (Zhang et al., 2022). Concurrent activation of *PI3K-AKT* and *HIF-1 signaling pathways* (**Figures 6F**, **9A**) suggests compensatory metabolic adaptations, such as glycolytic shifts, to sustain chronic inflammation.

The gut microbiota of LKP-treated mice exhibited severe dysbiosis, with Enterococcus faecium fermentation partially rescuing pathological symptoms (data were published in other paper). This highlights the interplay between fungal dysbiosis and bacterial communities in modulating host metabolism. *K. pintolopesii*’s ability to metabolize cholesterol and induce lipid droplet accumulation in macrophages (**Figure 5C**) may exacerbate foam cell formation and atherosclerotic-like changes, as observed in RA-associated metabolic syndrome. For instance, *K. pintolopesii* can suppress Candida albicans via secreted proteases, suggesting a competitive exclusion mechanism that could be leveraged therapeutically.

This study advances our understanding of how environmental fungi like *K. pintolopesii* disrupt immune homeostasis through multi-cellular crosstalk, metabolic reprogramming, and dysregulated cytokine networks. It elucidates how *K. pintolopesii* lysate triggers a cascade of immune-endothelial-fibroblast interactions that drive inflammatory arthritis and tissue fibrosis in genetically susceptible hosts. By unraveling these pathways, we highlight potential therapeutic targets to disrupt this deleterious network and ameliorate autoimmune pathology.

## Methods

### Lysate of *Kazachstania pintolopesii* prepared

Using Modified Martin’s Medium containing peptone 5.0g, Yeast extract powder 2.0g, glucose 20.0g, dipotassium hydrogen phosphate 1.0g, and magnesium sulfate 0.5g. The culture was incubated at 28°C on a shaker for 5 days, followed by centrifugation at 2000 rpm for 15 minutes, then collected the mycelium.

Mycelium is harvested, washed with cold PBS twice, resuspend in cold PBS. Mechanical grinding of the cell wall using a non-contact fully automatic ultrasonic cell disruption instrument (Bioruptor PICO). Protein concentration is determined via BCA or Bradford assay, and aliquots are stored at -80°C to prevent degradation. Key precautions include maintaining low temperatures, avoiding repeated freeze-thaw cycles, and optimizing buffer composition for downstream applications (e.g., avoiding strong detergents for enzyme activity assays).

### Fermentation products of *Enterococcus faecium* prepared

Using Modified Martin’s Medium containing peptone 5.0g, Yeast extract powder 2.0g, glucose 20.0g, dipotassium hydrogen phosphate 1.0g, and magnesium sulfate 0.5g. The culture was incubated at 28°C on a shaker for 7 days, followed by centrifugation at 2000 rpm for 15 minutes, then collected liquid supernatant.

Protein concentration is determined via BCA or Bradford assay, and aliquots are stored at -80°C to prevent degradation. Key precautions include maintaining low temperatures, avoiding repeated freeze-thaw cycles, and optimizing buffer composition for downstream applications (e.g., avoiding strong detergents for enzyme activity assays).

### Mice and treatment

All mouse procedures were approved by the Institutional Animal Care and Use committee of the Bo-Jin Biotechnology Co. LTD (Animal Ethics Application NO. BG-SMP-001V1-001). BALB/c ZAP70^W163C^ mutant mouse were purchased from the Changzhou Kalai Biotechnology Co. LTD [SCXK (Su) 2021-0013], then feeding and breeding in the Bo-Jin Biotechnology Co. LTD [SCXK (Yue) 2020-0051], and pair-housed in plastic cages in a temperature- controlled (25°C ± 2°C) colony room with a 12/12-h light/dark cycle. Food and water were available ad libitum. All efforts were made to minimize the number of animals used.

#### For lysate of *Kazachstania pintolopesii* treatments

20 male BALB/c ZAP70^W163C^ mutant mouse were divided into two group, 10 mice for every group. The lysate of *K. pintolopesii* treated group which administered via direct injection into the paravertebral muscle tissues of 0.2ml of lysate of *K. pintolopesii*, while the control group treated by PBS, once a week and last two months.

#### Fermentation products of *Enterococcus faecium* treatments

20 male BALB/c ZAP70^W163C^ mutant mouse were divided into two group, 10 mice for every group. The lysate of *E. faecium* treated group via intragastric administration of 0.5ml of lysate of *E. faecium*, while the control group treated by PBS, once a day and last two months.

### BMDM-ZAP70 sorting and culture

The basic culture protocol is as follows (1), Typically use 6-8-week-old BALB/c ZAP70^W163C^ mutant mice (2). High-glucose DMEM, supplemented with 10% FBS (Fetal Bovine Serum) and 1% Penicillin-Streptomycin (double antibiotics), added M-CSF (macrophage colony-stimulating factor) at 15% (v/v) (3). Bone marrow cell extraction, aseptically remove the femur and tibia from mice; thoroughly disinfect the bone surfaces by soaking in 75% ethanol (approximately 5 minutes, multiple times), then rinse with pre-cooled PBS to remove ethanol; cut off both ends of the bones. Flush the bone marrow cavity using a syringe (1–2 mL) filled with pre-cooled base medium to wash cells into a culture dish; repeatedly pipette the cell suspension to dissociate cell clumps, and filter through a 200-μm cell strainer to obtain a single-cell suspension; centrifuge (e.g., 1200 rpm, 5 minutes) to collect cells (4). Macrophage induction and differentiation, resuspend cells in complete DMEM containing 20–50 ng/mL M-CSF; seed the cells in culture dishes (bone marrow cells from one mouse can be seeded into 4-6 × 10 cm dishes); culture in a 37°C, 5% CO_2_ incubator, typically, add an equal volume of fresh induction medium on day 2.5 of culture; by approximately day 3, a large number of adherent macrophages (often with “small tail” morphology) can be observed, and cells are ready for experimentation on day 5 or 6.

### Isolation and ConA stimulation of C57BL/6 mouse splenocytes

#### Euthanasia and dissection

Sacrifice C57BL/6 mice by cervical dislocation. Disinfect the carcass by immersing in 75% alcohol for 5 minutes. Place the mouse on its back, make a small incision along the left ventral side (lower flank), and expose the spleen without puncturing it or allowing contact with fur. Remove the spleen carefully, trimming excess fat and connective tissue.

#### Cell extraction

Place two spleens onto a 40 μm cell strainer positioned over a 50 mL conical tube. Pre-wet the strainer with PBS. Gently grind the spleens using the flat end of a syringe plunger in a unidirectional motion. Rinse thoroughly with 5–10 mL PBS to maximize cell recovery. Centrifuge the suspension at 400–600×g for 5 minutes at 4°C. Discard the supernatant.

#### Red blood cell lysis

Resuspend the pellet in 2–5 mL of 1× RBC Lysis Buffer (e.g., ammonium chloride-based solution). Incubate on ice for 5–10 minutes (avoid exceeding 10 minutes to prevent leukocyte damage). Stop lysis by adding 10–20 mL of cold PBS. Centrifuge at 400-600 × g for 5 minutes at 4°C. Discard the supernatant.

#### Wash and resuspend

Resuspend the pellet in complete RPMI-1640 medium (RPMI-1640 + 10% FBS + 1% Penicillin-Streptomycin). Perform cell counting using a hemocytometer or automated counter. 5 × 10^8^ - 1 × 10^9^ cells per spleen. Centrifuge again at 400-600 × g for 5 minutes. Resuspend cells at 2-3 × 10^7^ cells/mL in complete RPMI-1640.

#### Culture setup

Prepare 5 flasks, each containing 18 mL of complete RPMI-1640. Add 10 μL of ConA (from a 1/2000 dilution stock) to 4 flasks. Leave one flask without ConA as a negative control. Distribute 2 mL of cell suspension (∼4 × 10^7^ cells) equally into each flask. Final density: ∼2 × 10^7^ cells/flask.

#### Culture maintenance

After 48 hours, observe medium acidification (yellow color). Add 20 mL of fresh complete RPMI-1640 (without ConA) to each flask to replenish nutrients. Harvest supernatant after 72 hours total culture.

#### Collection and storage

Centrifuge cultures at 200–300 × g for 10 minutes to pellet cells and debris. Collect the supernatant. Filter through a 0.22 μm filter if debris is present (optional but recommended for long-term storage). Store at –20°C or –80°C for future use.

This integrated protocol optimizes cell viability, minimizes debris, and ensures reproducible activation for downstream applications (e.g., cytokine analysis, immune cell assays).

### Isolation and culture of muscle stem cells

The isolation and culture of muscle stem cells (MuSCs) using the differential adhesion method involves digesting muscle tissue with enzymes like collagenase I/neutral protease II and trypsin substitute to create a single-cell suspension. This suspension is then subjected to sequential plating (e.g., PP1 to PP6), where faster-attaching cells like fibroblasts adhere first (within hours), allowing the slower-attaching MuSCs to be enriched from the supernatant and collected for further culture. The purified MuSCs are typically cultured in a serum-free medium supplemented with factors like recombinant epidermal growth factor and glutamine to promote proliferation and maintain stemness, often on surfaces coated with type I collagen to facilitate attachment. This approach effectively isolates Pax7-positive MuSCs capable of self-renewal and differentiation.

### Histopathology and immunostaining

Animal joint and spinal tissues were dissected. A total of four Joint and spinal tissues from each group were fixed in 4% paraformaldehyde solution and prepared as paraffin sections. Sections were stained with hematoxylin-eosin (H&E), and immunostained for IL-6 (Servicebio, GB11117, 1:600), IL-1β (Servicebio, GB11113, 1:1000), NF-κB (Servicebio, GB11997, 1:100), using paraffin-embedded 3 μm sections and a two-step peroxidase conjugated polymer technique (Servicebio, G1004, G1001, G1040, G1212, and G1202, Wuhan China; Beyotime, C0265, China). Slides were observed by light microscopy.

### Single-cell nuclear sequencing

#### Nuclei isolation sorting from joint and spinal tissues

Frozen Joint and spinal tissues were harvested and were washed in pre-cooled PBSE (PBS buffer containing 2 mM EGTA). Nuclei isolation was carried out using GEXSCOPE^®^ Nucleus Separation Solution (Singleron Biotechnologies, Nanjing, China) refer to the manufacturer’s product manual. Isolated nuclei were resuspended in PBSA mix Nuclei enriched in PBSA mix were stained with DAPI (1:1) (TermoFisher Scientifc, D1306). Nuclei were defined as DAPI-positive singlets.

#### Single nucleus RNA-sequencing library preparation

The concentration of single nucleus suspension was adjusted to 3-4 × 10^5^ nuclei/mL in PBSA mix. Single nucleus suspension was then loaded onto a microfluidic chip (GEXSCOPE^®^ Single Nucleus RNA-seq Kit, Singleron Biotechnologies) and snRNA-seq libraries were constructed according to the manufacturer’s instructions (Singleron Biotechnologies). The resulting snRNA-seq libraries were sequenced on an Illumina Novaseq 6000 instrument with 150 bp paired end reads.

#### Primary analysis of raw read data (snRNA-seq)

Raw reads were processed to generate gene expression profiles using CeleScope v1.5.2 (Singleron Biotechnologies) with default parameters. Briefly, Barcodes and UMIs were extracted from R1 reads and corrected. Adapter sequences and poly A tails were trimmed from R2 reads and the trimmed R2 reads were aligned against the GRCh38 (hg38) {GRCm38 (mm10)} transcriptome using STAR (v2.6.1b). Uniquely mapped reads were then assigned to genes with FeatureCounts (v2.0.1). Successfully Assigned Reads with the same cell barcode, UMI and gene were grouped together to generate the gene expression matrix for further analysis.

#### Quality control, dimension-reduction, and clustering (Seurat)

Seurat v 3.1.2 was used for quality control, dimensionality reduction and clustering (Wolf et al., 2018). For each sample dataset, we filtered expression matrix by the following criteria: 1) cells with gene count less than 200 or with top 2% gene count were excluded; 2) cells with top 2% UMI count were excluded; 3) cells with mitochondrial content > 5~20% were excluded; 4) genes expressed in less than 5 cells were excluded. After filtering, 9566 cells were retained for the downstream analyses, with on average 1473 genes and 2242 UMIs per cell. Gene expression matrix was normalized and scaled using functions NormalizeData and ScaleData. Top 2000 variable genes were selected by FindVariableFeatures for PCA analysis. Cells were separated into 9 clusters by FindClusters, using the top 50 principal components and resolution parameter at Louvain algorithm. Cell clusters were visualized using t-Distributed Stochastic Neighbor Embedding (t-SNE) or Uniform Manifold Approximation and Projection (UMAP) with Seurat functions RunTSNE and RunUMAP.

#### Batch effect removal

Harmony: Batch effect between samples was removed by Harmony v1.0 using the top 50 principal components from PCA (Butler et al., 2018).

CCA: Seurat’s CCA-based alignment was performed to obtain the batch-corrected space, and integration anchors were identified using top 20 principal components from PCA (Butler et al., 2018).

#### Differentially expressed genes analysis (Seurat)

To identify differentially expressed genes (DEGs), we used the Seurat FindMarkers function based on Wilcoxon rank sum test with default parameters, and selected the genes expressed in more than 10% of the cells in both of the compared groups of cells and with an average log2(Fold Change) value greater than 0.25 as DEGs. Adjusted p value was calculated by Bonferroni Correction and the value 0.05 was used as the criterion to evaluate the statistical significance.

#### Pathway enrichment analysis

To investigate the potential functions of different subpopulation cells, Gene Ontology (GO) and Kyoto Encyclopedia of Genes and Genomes (KEGG) analysis were used with the “clusterProfiler” R package v 3.16.1 (Yu et al., 2012). Pathways with p_adj value less than 0.05 were considered as significantly enriched. Selected significant pathways were plotted as bar plots. For GSVA pathway enrichment analysis, the average gene expression of each cell type was used as input data (Hanzelmann et al., 2013). Gene Ontology gene sets including molecular function (MF), biological process (BP), and cellular component (CC) categories were used as reference (Hanzelmann et al., 2013). Protein-protein interactions (PPI) of DEGs in xxx clusters were predicted based on known interactions of genes with relevant GO terms in the StringDB (1.22.0) (Szklarczyk et al., 2019).

#### Cell-type annotation

##### Cell-type recognition with Cell-ID

Cell-ID is multivariate approach that extracts gene signatures for each individual cell and performs cell identity recognition using hypergeometric tests (HGT) (Cortal et al., 2021). Dimensionality reduction was performed on normalized gene expression matrix through multiple correspondence analysis, where both cells and genes were projected in the same low dimensional space. Then a gene ranking was calculated for each cell to obtain most featured gene sets of that cell (Cortal et al., 2021). HGT were performed on these gene sets against joint and spinal tissues reference from SynEcoSys™ database, which contains all cell-type’s featured genes in the specific organ/tissue. Identity of each cell was determined as the cell-type has the minimal HGT p value. For cluster annotation, Frequency of each cell-type was calculated in each cluster, and cell-type with highest frequency was chosen as cluster’s identity.

The cell type identification of each cluster was determined according to the expression of canonical markers from the reference database SynEcoSys™ (Singleron Biotechnology). SynEcoSys™ contains collections of canonical cell type markers for single-cell seq data, from CellMakerDB, PanglaoDB and recently published literatures.

##### Subtyping of major cell types

To obtain a high-resolution map of subtyping of major cell types, cells from the specific cluster were extracted and reclustered for more detailed analysis following the same procedures described above and by setting the clustering resolution.

##### Filtering cell doublets

Cell doublets were estimated based on the expression pattern of canonical cell markers. Any clusters enriched with multiple cell type-specific markers were excluded for downstream analysis.

##### Filtering cell doublets and RNA contamination

To reduce the influence derived from RNA contamination and doublets in the downstream analysis, DecontX (Yang et al., 2020) was used to estimate and remove contamination, and DoubletFinder (McGinnis et al., 2019) was used to identify and remove doublet.

##### Cell cycle analysis

Cell Cycle score of each cell was calculated using the CellCycleScoring function implemented in the Seurat v 3.1.2 package (Hsiao et al., 2020).

#### ROE analysis

Ro/e denotes the ratio of observed to expected cell number in groups to measure the enrichment of cell types across different groups according the following formula: Ro/e=Observed/Expected. The expected cell number for cell types and groups are obtained from the chi-squared test (Zhang et al., 2018). If Ro/e > 1, it suggests that cells of the cell types are more frequently observed than random expectations in the specific groups, that is, enriched.

#### Cell-cell interaction analysis (CellCall)

CellCall v0.0.0.9000 (Zhang Y. et al., 2021) was used to analyze the intercellular interaction based on the receptor-ligand interaction between two cell types/subtypes, and inferred the signaling pathways of the internal regulation. The fraction of ligand-receptor genes interactions between cell types was assessed by integrating the L2 norm of the ligand-receptor interaction and the activity fraction of downstream TFs, which was calculated by the inbuilt GSEA algorithm. Finally, Ligand-receptor-TFs with a significant interaction between cell types was selected by using hypergeometric test and a p-value less than 0.05. Visualization was performed by using the inbuilt plotting functions from CellCall.

#### Cell-cell interaction analysis: CellPhoneDB

Cell-cell interaction (CCI) between different types were predicted based on known ligand–receptor pairs by Cellphone DB (v2.1.0) (Efremova et al., 2020) version. Permutation number for calculating the null distribution of average ligand-receptor pair expression in randomized cell identities was set to 1000. Individual ligand or receptor expression was thresholded by a cutoff based on the average log gene expression distribution for all genes across each cell type. Predicted interaction pairs with p value <0.05 and of average log expression > 0.1 were considered as significant and visualized by heatmap_plot and dot_plot in CellphoneDB.

#### Pseudotime trajectory analysis: monocle

Cell differentiation trajectory was reconstructed with Monocle3 v1.0.0 (Cao et al., 2019). Differentially expressed genes were used to sort cells in order of spatial-temporal differentiation. We used UMAP to perform graph_test and dimension-reduction and recognition trajectory by learn_graph function. Finally, the trajectory was visualized by plot_cells function.

### Subtyping of mononuclear phagocytes

To obtain a high-resolution map of Mononuclear phagocytes, cells from the specific cluster were extracted. Scanpy v1.9.3 was used for quality control, dimensionality reduction and clustering under Python 3.10. Top 2000 variable genes were selected by setting flavor = ‘seurat_v3’. Principle Component Analysis (PCA) was performed on the scaled variable gene matrix, and top 15 principle components were used for clustering and dimensional reduction. Cells were separated into 15 clusters by using Louvain algorithm and setting resolution parameter at 1.2. Cell clusters were visualized by using Uniform Manifold Approximation and Projection (UMAP). To annotate the cell clusters, DEGs were identified by the scanpy.tl.rank_genes_groups() function based on Wilcoxon rank sum test with default parameters. The cell groups were annotated based on the DEGs and the well-known cellular markers from the literature.

### RNA isolation and sequencing

NA concentration and purity were determined using a NanoDrop 2000 spectrophotometer (Thermo Fisher Scientific, Wilmington, DE, USA). RNA integrity was evaluated with the RNA Nano 6000 Assay Kit on an Agilent Bioanalyzer 2100 system (Agilent Technologies, Santa Clara, CA, USA), which provides an RNA Integrity Number (RIN) ranging from 1 to 10 to quantitatively assess sample quality.

A total of 1 μg RNA per sample was used as input for library preparation. Sequencing libraries were constructed using the NEBNext Ultra™ RNA Library Prep Kit for Illumina (New England Biolabs, Ipswich, MA, USA) in accordance with the manufacturer’s instructions. Unique index codes were incorporated to assign sequences to respective samples. Briefly, mRNA was enriched from total RNA using poly-T oligo-attached magnetic beads. Fragmentation was performed in the presence of divalent cations at elevated temperature using NEBNext First Strand Synthesis Reaction Buffer (5X). First-strand cDNA was synthesized with random hexamer primers and M-MuLV Reverse Transcriptase. Second-strand cDNA was subsequently generated using DNA Polymerase I and RNase H. Remaining overhangs were converted to blunt ends through exonuclease/polymerase activity. Following adenylation of the 3′ ends, NEBNext adaptors with hairpin loop structures were ligated to the cDNA fragments.

Library fragments were purified using the AMPure XP system (Beckman Coulter, Beverly, MA, USA) to select products of approximately 240 bp in length. The size-selected, adaptor-ligated cDNA was treated with 3 μL of USER Enzyme (NEB) at 37°C for 15 min, followed by incubation at 95°C for 5 min. PCR amplification was performed using Phusion High-Fidelity DNA Polymerase with universal PCR primers and index (X) primers. The PCR products were purified with the AMPure XP system, and library quality was validated on the Agilent Bioanalyzer 2100 system.

Cluster generation was conducted on a cBot Cluster Generation System using the TruSeq PE Cluster Kit v4-cBot-HS (Illumina). Finally, the libraries were sequenced on an Illumina platform to generate paired-end reads.

### Statistical analysis

Prism 8 and SPSS 16.0 software were used for statistical analyses. Significances between two groups were analyzed using two-tailed unpaired Student’s t-tests or Mann–Whitney U-tests. The significance of the growth curves was analyzed by two-way ANOVA. Survival curves were determined using the Kaplan–Meier method with the log-rank test. The data are presented as the mean ± SD except where stated otherwise. The differences with *p < 0.05, **p < 0.01, or ***p < 0.001 were considered statistically significant.

## Data Availability

The raw sequence data reported in this paper have been deposited in the Genome Sequence Archive (Chen et al., 2021) in National Genomics Data Center (Database resources of the national genomics data center, China national center for bioinformation in 2025, 2025), China National Center for Bioinformation/Beijing Institute of Genomics, Chinese Academy of Sciences (GSA: CRA030455 for primary spleen T cells; CRA030454 for bone marrow-derived macrophages and CRA030698 for single nuclear sequencing from BALB/c ZAP70^W163C^ mutant mice) that are publicly accessible at https://ngdc.cncb.ac.cn/gsa.

## Supplementary material

Supplementary Figure 1 The pathological examination of the hind limbs of the lysate of *Kazachstania pintolopesii* treated BALB/c ZAP70^W163C^ mutant mouse. Data are presented as means ± SD from more than three independent experiments. **p < 0.05 and* **p < 0.01 versus the model group, as determined by one-way ANOVA followed by the Holm-Šidák test.

Supplementary Figure 2 Immune cell interaction network changed in the Lysate of *K. pintolopesii* treated BALB/c ZAP70^W163C^ mutant mouse. Cell type specific markers of major cell types, T cells **(A)**, B cells **(B)**, Adipocytes **(C)**; Myocytes **(D)**; the major ligands and receptors of the interaction between Adipocytes and other 9 cell types **(E)**; Myocytes and other 9 cell types **(F)**; B cells and other 9 cell types **(G)**; RSS analysis showed the key genes who drive these changes. Data are presented as means ± SD from more than three independent experiments. **p < 0.05 and* **p < 0.01 versus the model group, as determined by one-way ANOVA followed by the Holm-Šidák test.

Supplementary Figure 3 Lysate of *K. pintolopesii* induces severe inflammatory response on the primary medullary macrophage of BALB/c ZAP70^W163C^ mutant mouse. Cell-cell communication through the Interactive CellChat Explorer showed that the interaction networks increased in the *K. pintolopesii* treated group **(B)** than that in the control group **(A)**; Heatmap of differential expression of transcription factors of each macrophage subpopulations **(C)**; KEGG pathway of down-regulated genes enrich analysis **(D)**. Data are presented as means ± SD from more than three independent experiments. **p < 0.05 and* **p < 0.01 versus the model group, as determined by one-way ANOVA followed by the Holm-Šidák test.

Supplementary Figure 4 Lysate of *K. pintolopesii* induces severe inflammatory response on the primary medullary macrophage of BALB/c ZAP70^W163C^ mutant mouse. The changes of transcription were detected using RNA-seq **(A)**, the proinflammatory cytokines of IL-1β, IL-6, IL-7, IL-18, TNF-α were sharply up-regulated **(C, D)**, IFNs were activated in the LKP-treated BMDM-ZAP70 **(B)**. Data are presented as means ± SD from more than three independent experiments. **p < 0.05 and* **p < 0.01 versus the model group, as determined by one-way ANOVA followed by the Holm-Šidák test.

Supplementary Figure 5 Lysate of *K. pintolopesii* promote the fibroblastlike synoviocyte abnormal proliferation in the Lysate of *K. pintolopesii* treated BALB/c ZAP70^W163C^ mutant mouse. KEGG pathway enrichment analysis of differentially expressed genes (DEGs) in osteoblast subpopulations from *K. pintolopesii*-treated mice, assessed via single-nucleus RNA sequencing, is presented for Fib_*Acan***(D)**, Fib_*Celf2***(C)**, Fib_*Cmss1***(B)**, and Fib_*Tnc***(A)**. Data are presented as means ± SD from more than three independent experiments. **p < 0.05 and* **p < 0.01 versus the model group, as determined by one-way ANOVA followed by the Holm-Šidák test.

Supplementary Figure 6 Lysate of *K. pintolopesii* influence the endothelial cells significant changed in the Lysate of *K. pintolopesii* treated BALB/c ZAP70^W163C^ mutant mouse. KEGG pathway enrichment analysis of differentially expressed genes (DEGs) in osteoblast subpopulations from *K. pintolopesii*-treated mice, assessed via single-nucleus RNA sequencing, is presented for AECs_*Mgp***(A)** VECs_*Col15a1***(C)**, VECs_*Mcam***(D)** and VECs_*Vwf***(E)**. Data are presented as means ± SD from more than three independent experiments. **p < 0.05 and* **p < 0.01 versus the model group, as determined by one-way ANOVA followed by the Holm-Šidák test.
