# Supplementary figures and images for: Kazachstania pintolopesii triggers an immune-endothelial-fibroblast cascade and drives inflammatory arthritis and tissue fibrosis in genetically susceptible hosts
Source: Front Cell Infect Microbiol. 2025 Dec 11;15:1738184. doi: 10.3389/fcimb.2025.1738184 (PMC12738364; doi:10.3389/fcimb.2025.1738184)

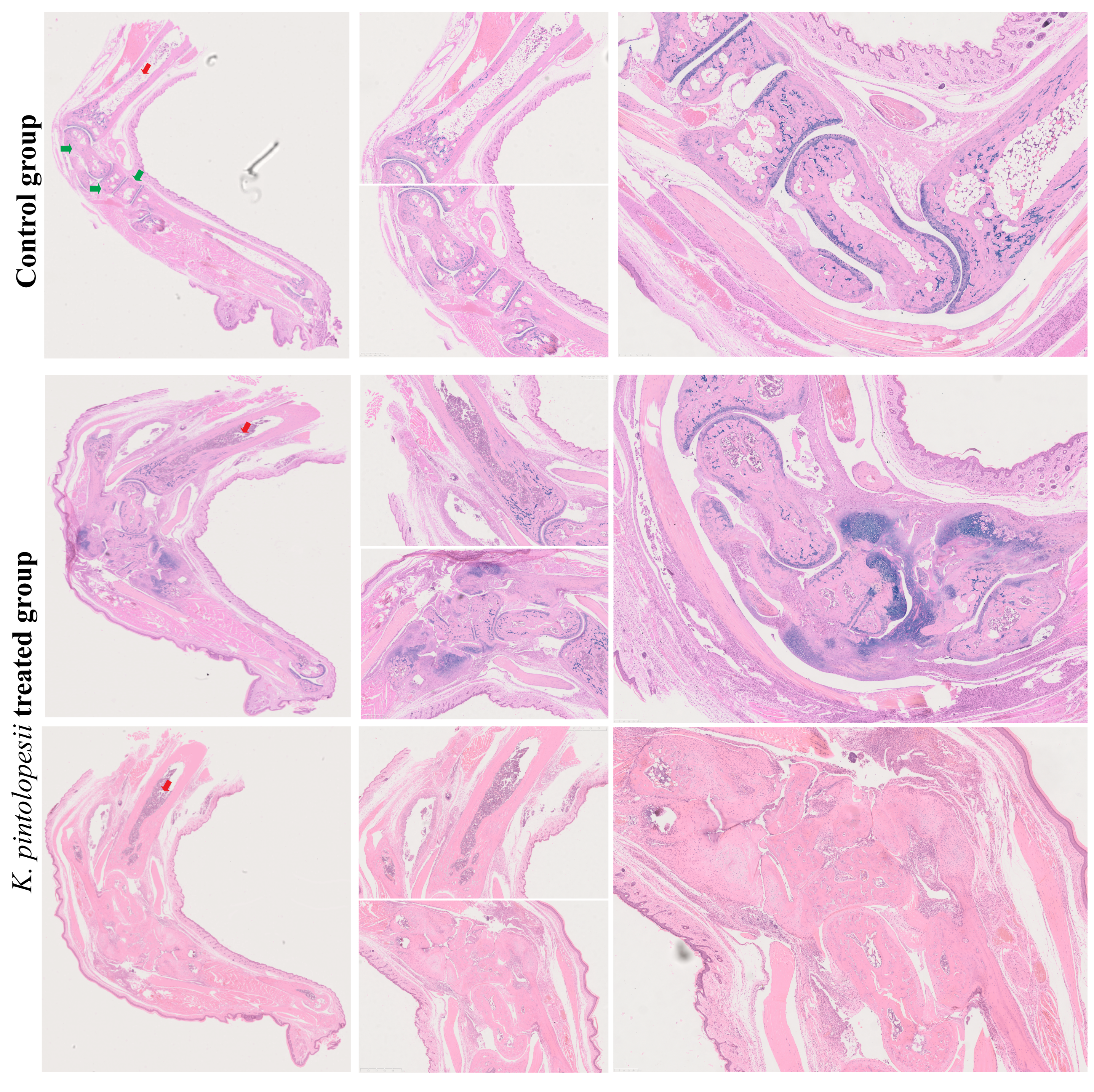

Supplement: Supplementary Figure 1 — The pathological examination of the hind limbs of the lysate of Kazachstania pintolopesii treated BALB/c ZAP70W163C mutant mouse. Data are presented as means ± SD from more than three independent experiments. *p < 0.05 and **p < 0.01 versus the model group, as determined by one-way ANOVA followed by the Holm-Šidák test. [file Image1.tif]

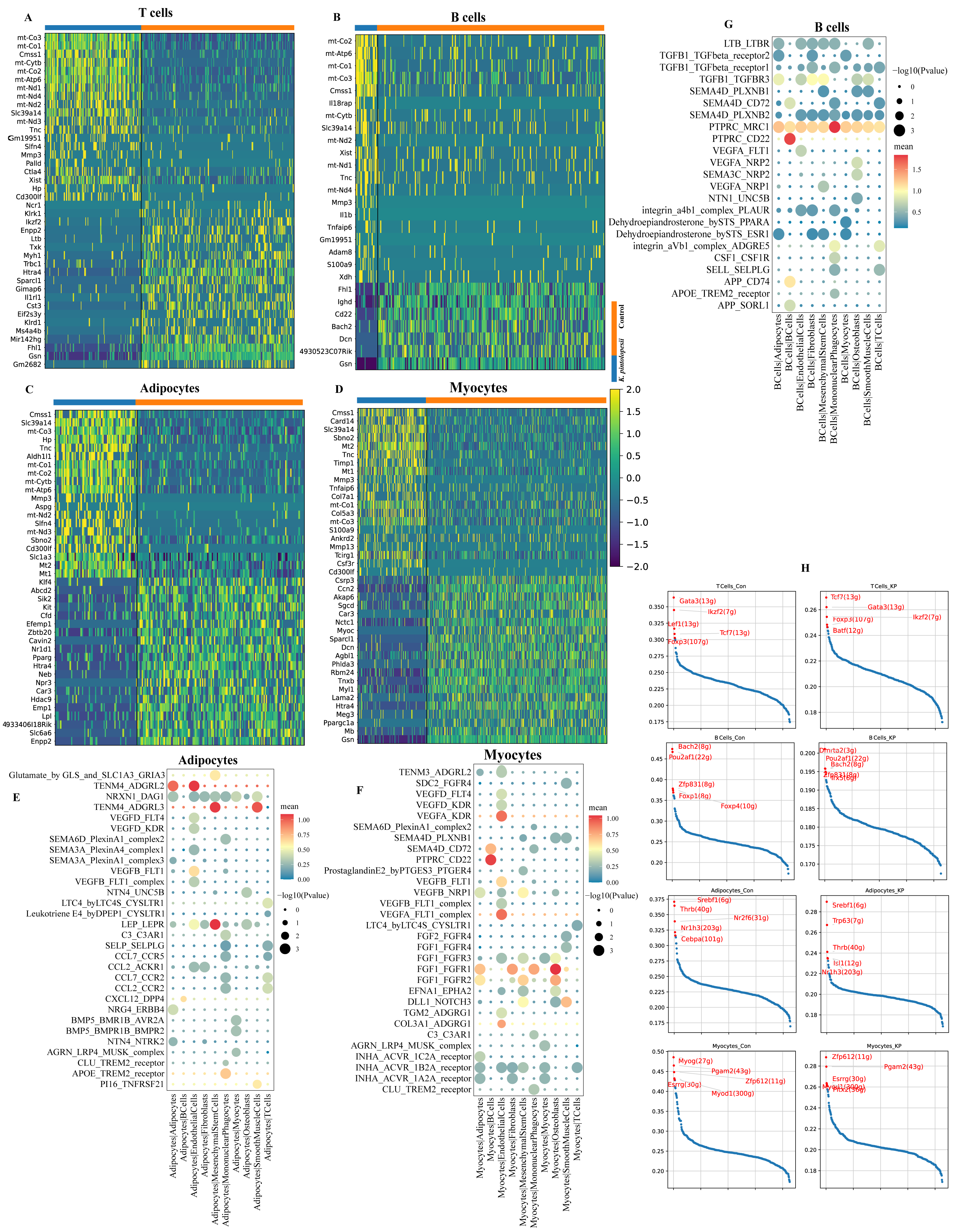

Supplement: Supplementary Figure 2 — Immune cell interaction network changed in the Lysate of K. pintolopesii treated BALB/c ZAP70W163C mutant mouse. Cell type specific markers of major cell types, T cells (A), B cells (B), Adipocytes (C); Myocytes (D); the major ligands and receptors of the interaction between Adipocytes and other 9 cell types (E); Myocytes and other 9 cell types (F); B cells and other 9 cell types (G); RSS analysis showed the key genes who drive these changes. Data are presented as means ± SD from more than three independent experiments. *p < 0.05 and **p < 0.01 versus the model group, as determined by one-way ANOVA followed by the Holm-Šidák test. [file Image2.tif]

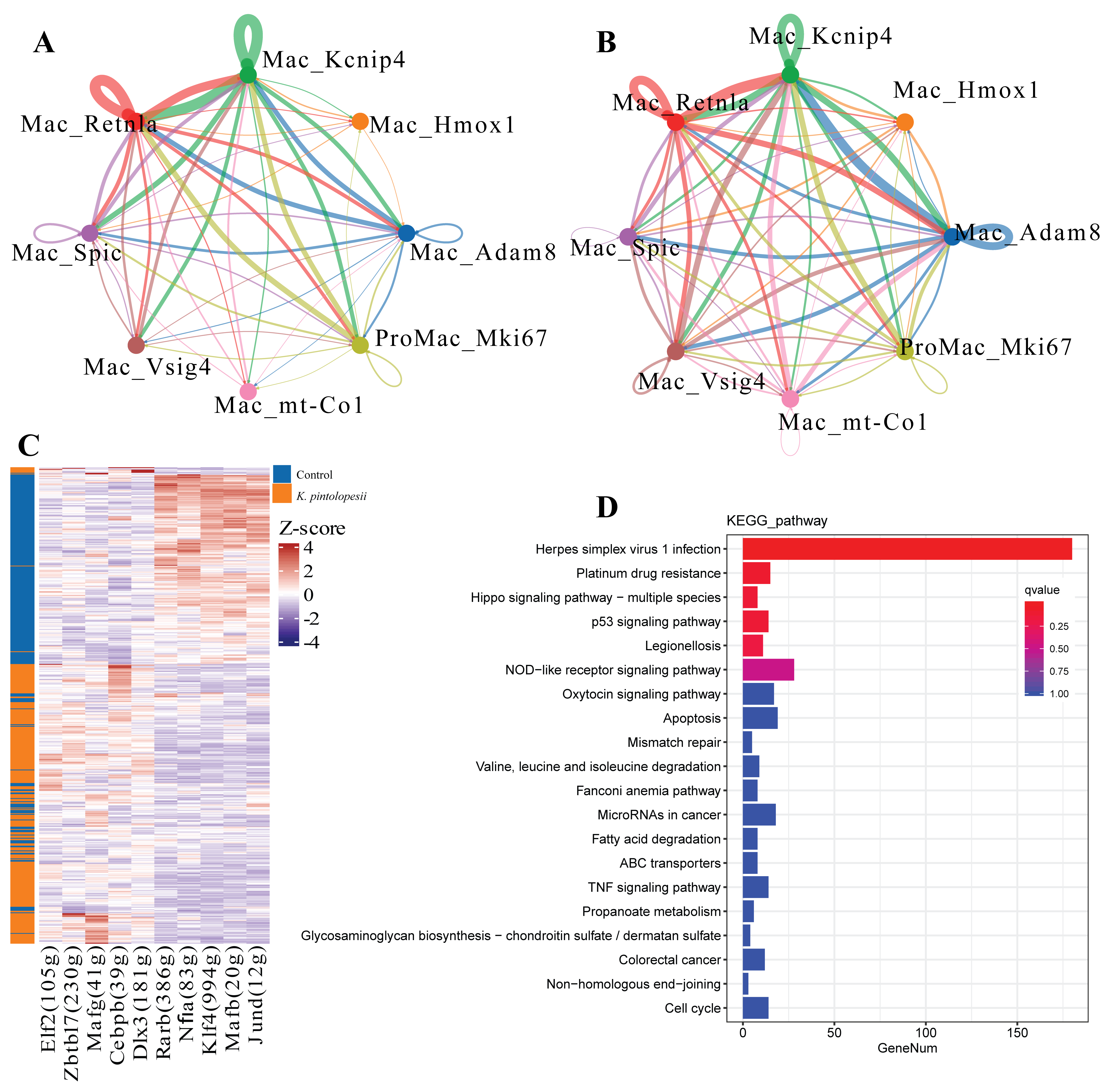

Supplement: Supplementary Figure 3 — Lysate of K. pintolopesii induces severe inflammatory response on the primary medullary macrophage of BALB/c ZAP70W163C mutant mouse. Cell-cell communication through the Interactive CellChat Explorer showed that the interaction networks increased in the K. pintolopesii treated group (B) than that in the control group (A); Heatmap of differential expression of transcription factors of each macrophage subpopulations (C); KEGG pathway of down-regulated genes enrich analysis (D). Data are presented as means ± SD from more than three independent experiments. *p < 0.05 and **p < 0.01 versus the model group, as determined by one-way ANOVA followed by the Holm-Šidák test. [file Image3.tif]

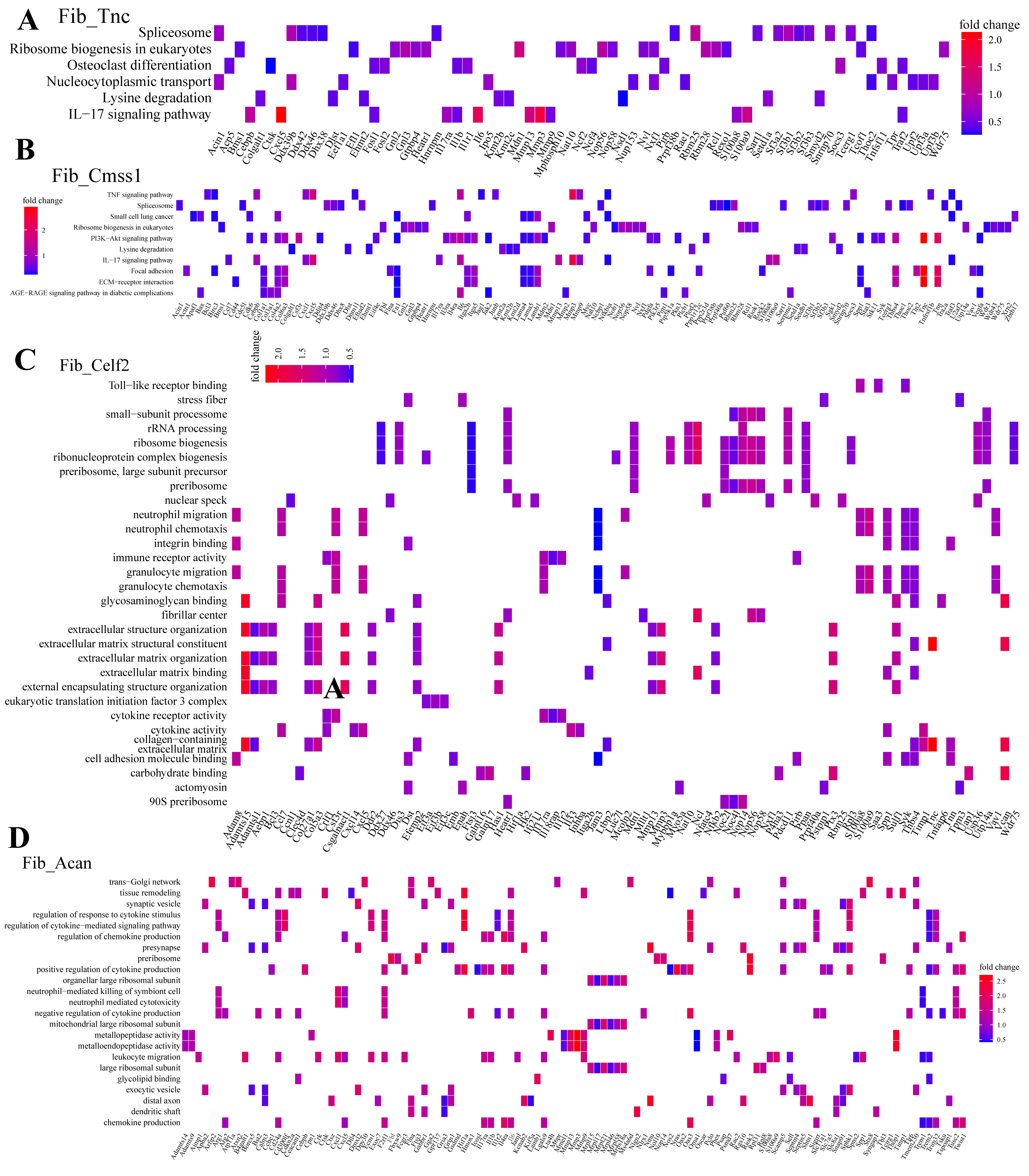

Supplement: Supplementary Figure 5 — Lysate of K. pintolopesii promote the fibroblastlike synoviocyte abnormal proliferation in the Lysate of K. pintolopesii treated BALB/c ZAP70W163C mutant mouse. KEGG pathway enrichment analysis of differentially expressed genes (DEGs) in osteoblast subpopulations from K. pintolopesii-treated mice, assessed via single-nucleus RNA sequencing, is presented for Fib_Acan(D), Fib_Celf2(C), Fib_Cmss1(B), and Fib_Tnc(A). Data are presented as means ± SD from more than three independent experiments. *p < 0.05 and **p < 0.01 versus the model group, as determined by one-way ANOVA followed by the Holm-Šidák test. [file Image5.tif]

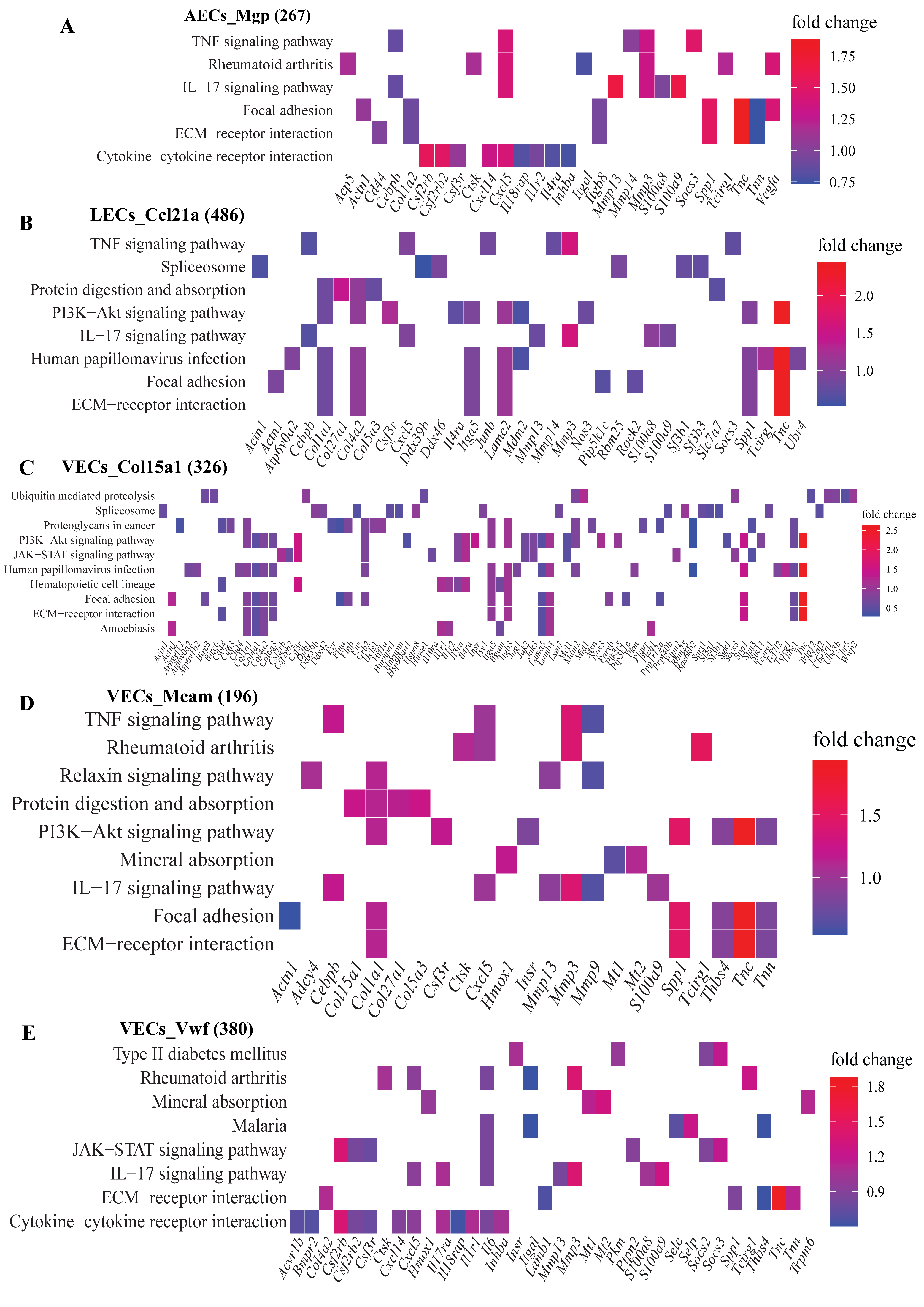

Supplement: Supplementary Figure 6 — Lysate of K. pintolopesii influence the endothelial cells significant changed in the Lysate of K. pintolopesii treated BALB/c ZAP70W163C mutant mouse. KEGG pathway enrichment analysis of differentially expressed genes (DEGs) in osteoblast subpopulations from K. pintolopesii-treated mice, assessed via single-nucleus RNA sequencing, is presented for AECs_Mgp(A) VECs_Col15a1(C), VECs_Mcam(D) and VECs_Vwf(E). Data are presented as means ± SD from more than three independent experiments. *p < 0.05 and **p < 0.01 versus the model group, as determined by one-way ANOVA followed by the Holm-Šidák test. [file Image6.tif]
